# 3-Ethynyltriimidazo[1,2-*a*:1′,2′-*c*:1″,2″-*e*][1,3,5]triazine Dual Short- and Long-Lived Emissions with Crystallization-Enhanced Feature: Role of Hydrogen Bonds and π-π Interactions

**DOI:** 10.3390/molecules29091967

**Published:** 2024-04-25

**Authors:** Daniele Malpicci, Daniele Maver, Elisabetta Rosadoni, Alessia Colombo, Elena Lucenti, Daniele Marinotto, Chiara Botta, Fabio Bellina, Elena Cariati, Alessandra Forni

**Affiliations:** 1 Department of Chemistry, Università degli Studi di Milano, Via Golgi 19, 20133 Milano, Italy; daniele.malpicci@unimi.it (D.M.); daniele.maver@unimi.it (D.M.); alessia.colombo@unimi.it (A.C.); 2 Institute of Chemical Sciences and Technologies “Giulio Natta” (SCITEC) of CNR, Via Golgi 19, 20133 Milano, Italy; elena.lucenti@scitec.cnr.it (E.L.); daniele.marinotto@scitec.cnr.it (D.M.); 3 Department of Chemistry and Industrial Chemistry, University of Pisa, Via Moruzzi 13, 56124 Pisa, Italyfabio.bellina@unipi.it (F.B.); 4 INSTM Research Unit of Milano, Via Golgi 19, 20133 Milano, Italy; 5 Institute of Chemical Sciences and Technologies “Giulio Natta” (SCITEC) of CNR, Via Corti 12, 20133 Milano, Italy; chiara.botta@scitec.cnr.it

**Keywords:** organic room temperature phosphorescence, dual emission, crystallization-enhanced emission, supramolecular interactions

## Abstract

Organic room temperature phosphorescent (ORTP) materials with stimuli-responsive, multicomponent emissive behaviour are extremely desirable for various applications. The derivative of cyclic triimidazole (**TT**) functionalized with an ethynyl group, **TT-CCH**, is isolated and investigated. The compound possesses crystallization-enhanced emission (CEE) comprising dual fluorescence and dual phosphorescence of both molecular and supramolecular origin with aggregation-induced components highly sensitive to grinding. The mechanisms involved in the emissions have been disclosed thanks to combined structural, spectroscopic and computational investigations. In particular, strong CH⋯N hydrogen bonds are deemed responsible, for the first time in the **TT** family, together with frequently observed π⋯π stacking interactions, for the aggregated fluorescence and phosphorescence.

## 1. Introduction

Single-component organic materials characterized by rich emissive behaviour, including room-temperature long-lived features, are receiving growing attention from the scientific community due to their benefits when compared to widely used metal-containing compounds, including biocompatibility and low cost. Applications of organic room-temperature phosphorescence (ORTP) in different fields spanning bioimaging [1,2,3,4], anti-counterfeiting [5,6,7,8,9,10,11], sensing [12] and displays [13] have been assessed.

However, most organic materials are characterized by weak spin–orbit coupling, and, generally, ORTP is prevented by prevailing non-radiative transitions of triplet excitons caused by molecular motions and oxygen. Many strategies have been developed to overcome these drawbacks. On the one hand, the introduction of suitable functional groups (e.g., carbonyls, heavy atoms, and heteroatoms) could enhance spin–orbit coupling and, therefore, promote the singlet-to-triplet intersystem crossing. On the other hand, the suppression of the non-radiative decays of triplet excitons has been realized through the establishment of a rigid molecular environment based on intermolecular interactions, such as stacking interactions [14,15,16,17,18,19,20] and hydrogen [21,22] and halogen bonding [23,24,25], host–guest systems [26,27,28,29,30,31,32,33], co-assembly based on macrocyclic compounds [30], crystallization [34,35,36], and cocrystallization [37,38], doping in a polymer matrix [39,40,41]. The mechanisms involved in the photophysics of multi-emissive ORTP systems have been clarified through combined spectroscopic, structural, and computational studies. Interestingly, frequently, intermolecular interactions are able not only to stabilize emitting states but also to generate supramolecular structures with additional radiative deactivation channels [42,43].

In the last few years, our group has been involved in the preparation and characterization of various members of a family of compounds with triimidazo[1,2-*a*:1′,2′-*c*:1″,2″-*e*][1,3,5]triazine, **TT** [18], as a prototype. Many **TTs** have been revealed not only as interesting ORTP materials but also as ligands for the construction of photoluminescent complexes and coordination polymers. One peculiar feature of many **TTs** and of **TT** itself is an excitation-dependent PL (photoluminescent) behaviour comprising an ultralong phosphorescence associated with the presence of strong π-π stacking interactions in the crystalline structure. In this regard, we recently isolated and characterized 3-(9*H*-carbazol-9-yl)triimidazo[1,2-*a*:1′,2′-*c*:1″,2″-*e*][1,3,5]triazine, TT-(N)-Cz, which crystallizes in two polymorphs (TT-(N)-CzM and TT-(N)-CzT), showing structure-dependent dual fluorescence (high-energy fluorescence, HEF, and low-energy fluorescence, LEF) and dual phosphorescence (high-energy phosphorescence, HEP, and low-energy phosphorescence, LEP) [44]. Thanks to combined X-ray diffraction (XRD) analysis, DFT/TDDFT calculations and spectroscopic investigations, the origin of the mechano-responsive LEF and LEP has been attributed to aggregated excited singlet and triplet states of π-π **TT** character. Importantly, TT-(N)-Cz represents the first example among TTs so far isolated, also showing, in addition to a long component (LEP), a fast (LEF) emission with an aggregated origin [44].

**Scheme 1:**
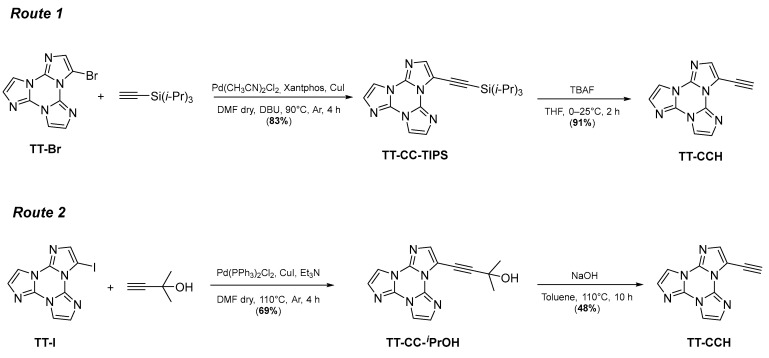
Synthesis of TT-CCH.

Herein, we describe the synthesis, structural and extended photophysical characterization of 3-ethynyltriimidazo[1,2-*a*:1′,2′-*c*:1″,2″-*e*][1,3,5]triazine, **TT-CCH** (Scheme 1), which displays enhanced properties in its aggregates rather than as a molecule according to a CEE (crystallization-enhanced emissive) behaviour, resulting in dual fluorescence and dual phosphorescence. The origin of these emissive features is disclosed through X-ray diffraction analysis and DFT/TDDFT calculations and assigned to both molecular and supramolecular entities, the latter associated, for the first time among **TTs**, with the formation of strong CH⋯N hydrogen bonds, in addition to the frequently observed π⋯π stacking interactions.

## 2. Results

**TT-CCH** has been prepared according to two different synthetic routes (Scheme 1) involving Sonogashira reaction of either **TT-Br** with (triisopropylsilyl)acetylene followed by desilylation with tetrabutylammoniumfluoride, TBAF, (Route 1) or **TT-I** with 2-methylbut-3-yn-2-ol and further deprotection under basic conditions (Route 2) [45].

### 2.1. Photophysical Characterization

Batches of **TT-CCH** obtained from both synthetic routes have been purified by flash chromatography and repeated crystallizations from DCM/MeOH before their photophysical characterization to exclude impurities concerns. Superimposable results have been obtained for all samples.

**Figure 1:**
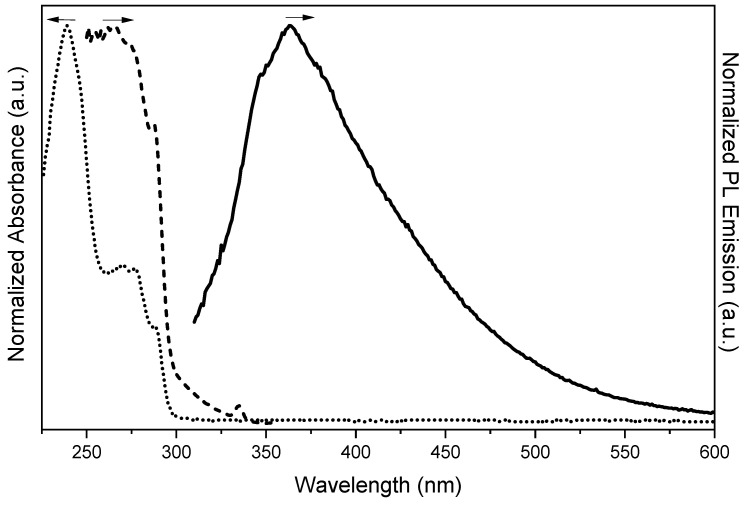
Absorption (dotted line), excitation (dashed line, λ_em_: 374 nm) and emission (continuous line, λ_exc_: 290 nm) spectra of **TT-CCH** in DCM.

In DCM solutions (5 × 10^−5^ M), **TT-CCH** displays an intense absorption band at 240 nm, followed by a less intense one at 270 nm with a shoulder at 287 nm. In the same conditions, the PL spectrum exhibits, by exciting at 290 nm, a single, broad fluorescence at about 360 nm (Figure 1 and Table 1; lifetime, τ, below instrumental resolution) with photoluminescent quantum yield, Φ, equal to 2%. The corresponding excitation profile perfectly overlaps with the absorption spectrum, showing, in particular, a low energy component at 287 nm. At lower concentrations (1 × 10^−5^ M), the Raman signal of the solvent is comparable with the fluorescence of the compound, testifying to a drastic decrease in the emission intensity (see Figure S9).

**Figure 2:**
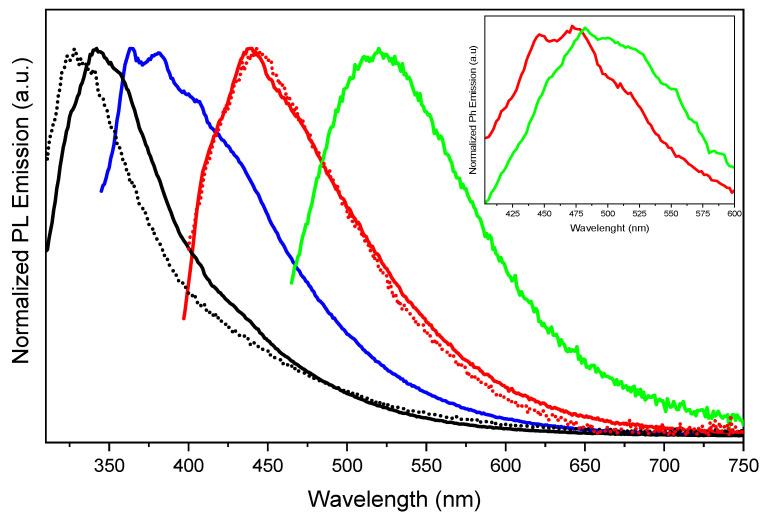
Emission spectra of **TT-CCH** in PMMA matrix (0.1 *w*/*w*%, dotted line; 5 *w*/*w*%, full line) at 298 K. λ_exc_: 290 nm (black lines), 330 nm (blue line), 380 nm (red lines) and 445 nm (green line). Inset: The delayed spectra are shown for the 5 *w*/*w*% concentration at short (40 µs delay, 200 µs window; red line) and long (4 ms delay, 20 ms window; green line) delay times.

In PMMA-blended films, an excitation and dye/matrix loading-dependent emissive behaviour is observed. Spectra obtained for dye/matrix *w*/*w*% equal to 0.1 and 5 are reported in Figure 2 (results for the 1.5 *w*/*w*% can be found in Figure S10). When excited at 290 nm, the 0.1% sample displays a single, unstructured fluorescence, HEF, at 330 nm (τ shorter and Φ lower than the instrumental detection limits). However, a broad phosphorescence at 442 nm, HEP, can be selectively activated by exciting at sufficiently low energy (380 nm) to exclude the otherwise stronger fluorescence. By increasing the dye loading, a more complex, excitation-dependent emissive behaviour is observed together with an intensification of the quantum efficiency (Φ = 3% for the 5 *w*/*w*% film). In particular, by exciting at 290 nm, a red-shifted HEF dominates the PL spectrum (centred at about 342 nm for the 5 *w*/*w*% film and 338 nm for the 1.5% *w*/*w*% sample). Such red shifting can be explained by the presence of an additional lower-energy fluorescence, LEF (centred at about 383 nm for both the 5 and 1.5 *w*/*w*% loadings), which becomes the predominant component in the spectrum at lower-energy excitation (330 nm). Moreover, HEP at 442 nm can be activated by exciting the films at 380 nm as already reported for the 0.1 *w*/*w*%, but for films with higher dye content, an additional long-lived component at lower energy, LEP, centred at about 522 nm is observed when monitoring the PL spectrum at 445 nm excitation. In the inset of Figure 2, the phosphorescence spectra of **TT-CCH** in the PMMA matrix at 5% *w*/*w*% at different delays are reported. The spectrum at a shorter time delay, with peaks at 445 and 473 nm, corresponds well to HEP. By increasing the time delay, the contribution from the longer-lived LEP at 550 nm is observed. This result indicates that aggregates are present in the 5 *w*/*w*% PMMA film. Conversely, the same measurements performed on the 0.1 *w*/*w*% PMMA film do not show the 550 nm contribution (see Figure S11).

**Figure 3:**
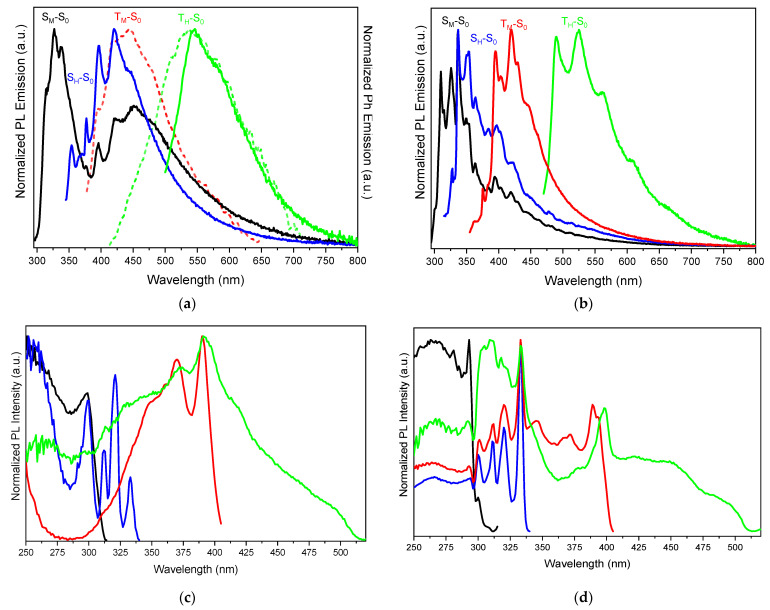
Normalized emission and excitation spectra of **TT-CCH** crystals at 298 K (**a**,**c**) and 77 K (**b**,**d**). (**a**) Prompt (full line) and delayed (dashed line) emission spectra; λ_exc:_: 275 nm (black line); 330 nm (blue line) and 486 nm (green line); λ_exc:_: 310 nm (dashed red, delay 15 µs, window 400 µs; dashed green, delay 1 ms, window 15 ms). (**b**) Prompt emission spectra; λ_exc:_: 275 nm (black line); 300 nm (blue line); λ_exc:_: 340 nm (red line); λ_exc:_: 450 nm (green line). (**c**,**d**) Excitation spectra: λ_em:_: 326 nm (black line); 354 nm (blue line); 420 nm (red line) and 550 nm (green line).

**Figure 4:**
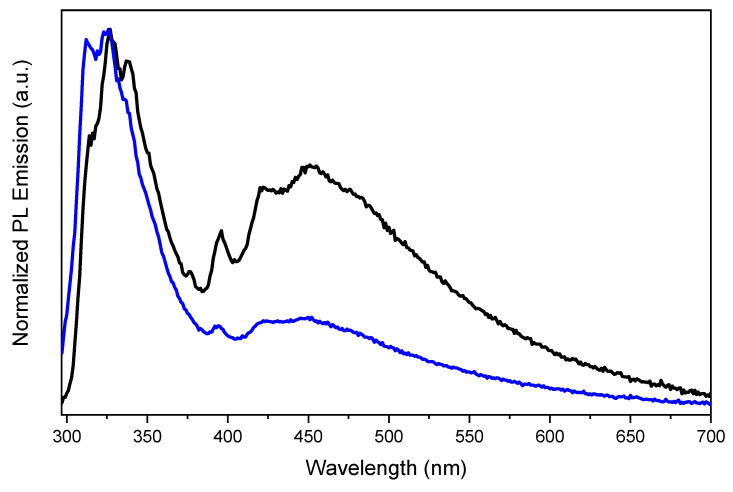
Emission spectra of **TT-CCH** crystals at 298 K before (black line) and after (blue line) manual grinding in a mortar; λ_exc:_: 275 nm.

Similar to what was observed in PMMA-blended films at high dye-loading, crystals of **TT-CCH** (Φ = 16%, Figure 3 and Table 1) reveal an excitation-dependent PL behaviour comprising two fluorescences and two phosphorescences. Specifically, with regards to the fluorescence contributions, a high-energy fluorescence (HEF) with maxima at 314, 326, 338 nm (τ below instrumental resolution) and a low-energy fluorescence (LEF) at 354, 367, 377 nm (τ = 1.05 ns, Figure S16) are recognizable in the spectra when exciting at high energy (λ_exc_ = 275–330 nm). The relative intensity of the two components is temperature/excitation dependent, with LEF being weaker than (and almost overwhelmed by) HEF at room temperature but becoming comparable at 77 K when excited at 275 nm. The corresponding excitation profiles display at 298 K a broad signal with a maximum at 299 nm for HEF, slightly red-shifted with respect to the low energy peak observed in DCM solution (287 nm), and a vibronic envelope at 311, 320, 333 nm for LEF. The excitation replicas of LEF are preserved at 77 K, while the HEF excitation profile is split into a major narrow peak centred at 293 nm with a much weaker component at 300 nm. Moreover, at 275 nm excitation, the relative intensity of HEF and LEF vary by manually grinding, in a mortar, crystals of **TT-CCH** (Figure 4). In particular, after grinding, the HEF high-energy vibronic peak at 314 nm becomes stronger than the HEF/LEF-superimposed lower-energy replicas, and the LEF peculiar signal at 377 nm is attenuated.

Concerning the long-lived components, a high-energy phosphorescence (HEP) and a low-energy one (LEP) appear in the PL spectrum with maxima at room temperature, at 396, 420 nm (τ = 0.25 ms, Figure S17) and 545 nm (τ = 4.66 ms, Figure S18), respectively. Partially resolved vibrational components at 370 and 390 nm are present in the excitation profile of HEP, while less resolved broad peaks at about 395, 415, 450 nm are observed by monitoring LEP both at 298 and 77 K. Interestingly, while LEP is visible only by exciting at sufficiently low energy (about 450 nm) and is otherwise overwhelmed by the stronger fluorescences, HEP is clearly visible, together with HEF, already at high energy excitation. The two phosphorescences are recognizable at 434 (HEP) and 538 (LEP) nm in Ph spectra (Figure 3a) by exciting at 310 nm at 15 µs and 1 ms delays, respectively. At 77 K HEP and LEP, a better vibronic resolution is shown (Figure 3b). Moreover, both long-lived contributions appear strongly attenuated, with respect to HEF, by manual grinding (Figure 4).

Overall, the results obtained in the DCM solution, PMMA-blended films and powders concur to suggest HEF and HEP as due to molecular entities and LEF and LEP deriving from supramolecular ones. More specifically, (i) aggregation concerns due to the low solubility of the compound in a common organic solvent, together with its low quantum efficiency, do not allow the disclosure of the origin of the broadband observed in the DCM solution. In fact, this emission could be the result of the superimposition of HEF and LEF. (ii) In PMMA films, LEF and LEP appear only by increasing the dye-loading; (iii) the mechanochromic dependence of the relative intensity of HEF and LEF suggests a supramolecular origin of the latter. For what concerns HEP and LEP, both emissions are sensitive to the sample crystallinity degree, a behaviour commonly observed for the long-lived emissions of the **TT** family [18,44,46]; in fact, rigidification and protection from molecular oxygen by the crystal framework positively affect phosphorescences. However, only the LEP position corresponds to that observed for members of the **TT** family characterized by strong π-π stacking interactions of the **TT** ring and, therefore, usually attributed to aggregation phenomena. The molecular origin of HEP is supported also by its appearance in low-loaded PMMA films.

### 2.2. Structural Characterization

**Figure 5:**
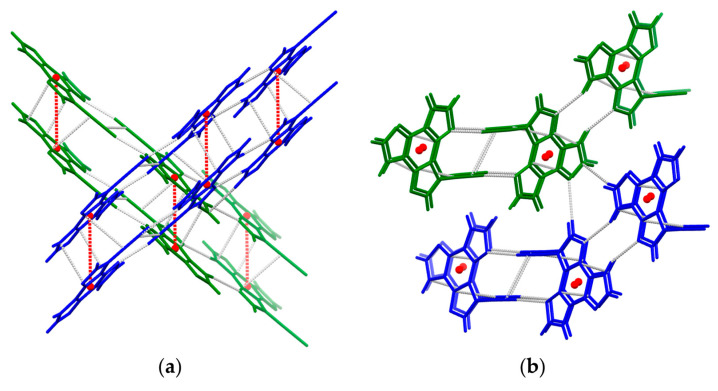
Fragment of the crystal packing of **TT-CCH** with two stacked ribbons drawn in blue and green, respectively, evidencing (**a**) the π⋯π stacking motif and (**b**) the hydrogen-bonded pattern within and between ribbons (red spheres represent the triazinic geometrical centroids; C⋯C and H⋯N intermolecular contacts shorter than the sum of vdW radii are plotted with grey dashed lines).

To disclose the nature of intermolecular interactions governing the aggregated phase of **TT-CCH**, X-ray diffraction studies have been conducted on single crystals obtained from DCM/MeOH solutions (Table S1 and Figure 5). The compound crystallizes in the monoclinic P2_1_/c space group in the form of elongated colourless blocks. The crystal structure is governed by the formation of infinite ribbons of essentially coplanar centrosymmetry-related **TT-CCH** molecules, connected to each other through quite short CH⋯N hydrogen bonds (HBs) forming cyclic patterns (r_H⋯N_ = 2.33 and 2.48 Å, the former involving the acidic C(*sp*)–H bond). The ribbons are laterally connected to the adjacent, almost perpendicular, ones (dihedral angle between **TT** l.s. planes equal to 89°) through weaker CH⋯N HBs. In the third direction, they form columnar π⋯π aggregates with interplanar distance equal to 3.248 Å and distance between triazinic geometrical centroids equal to 4.635 Å (the shorter C⋯C contacts are C9⋯C8*_x,_*_1*+y,z*_, 3.360(2) Å and C3⋯C6*_x,_*_1*+y,z*_, 3.393(2) Å). It can, therefore, be asserted that strong CH⋯N and π⋯π intermolecular interactions are established in the solid state, with the latter being normally observed in the crystal structure of several members of the **TT** family [17,18,44,46,47,48,49,50,51,52,53].

### 2.3. Computational Studies

DFT and TDDFT calculations have been performed to investigate the possible role played by the strong intermolecular interactions in activating the additional fluorescence and phosphorescence observed at low energy (LEF and LEP) in the aggregated phases. Both **TT-CCH** isolated molecules and several small aggregates, comprising CH⋯N and/or π⋯π stacked dimers and tetramers, have been taken into consideration. The simulated absorption spectrum of the isolated molecule (Figures S20 and S21 and Table S2) reproduces the UV one well, though it is shifted at slightly higher energy; an intense band is predicted at 205 nm as the result of the convolution of several high energy states (in particular S_6_, having oscillator strength, *f*, equal to 0.51), which corresponds to the intense 240 nm UV band. The two lower-energy states, S_1_ and S_2_, computed at 242 (*f* = 0.25) and 225 nm (*f* = 0.15), correspond to the weaker absorptions at 287 and 270 nm, respectively. S_1_ is essentially (81%) a HOMO→LUMO ^1^(π,π*) transition involving the C≡C bond and the portion of the TT moiety directly bonded to it (Figure S24). Close to S_1_ and almost overlapped to it, a ^3^(π,π*) triplet state (T_6_) is computed with ΔE_S1-T6_ < 0.001 eV. This triplet state is a combination of several contributions besides HOMO and LUMO, including orbitals (HOMO-1 and LUMO+1) which are delocalized only on **TT**. The insignificant energy gap between S_1_ and T_6_, together with the partially different delocalization scheme of the two states, both factors favouring the singlet-to-triplet intersystem crossing, ISC, explain the intense molecular phosphorescence observed at about 400 nm and interpreted as radiative deactivation from the T_1_^3^(π,π*) state reached through internal conversion, IC, from T_6_.

**Figure 6:**
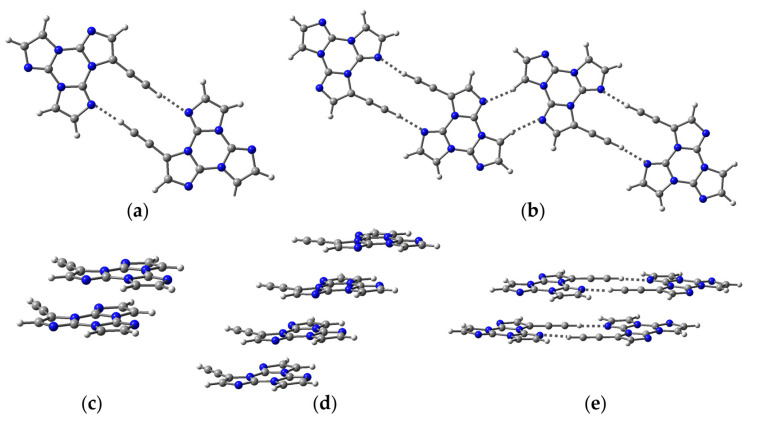
ωB97X/6-311++G(d,p) optimized aggregates of **TT-CCH**: (**a**) H-bonded dimer; (**b**) H-bonded tetramer; (**c**) π-π stacked dimer; (**d**) π-π stacked tetramer; (**e**) tetramer comprising two π-π stacked H-bonded dimers. Atom color scheme: C, gray; H, light gray; N, blue.

DFT optimizations of a series of CH⋯N and/or π⋯π stacked dimers and tetramers (Figure 6), starting from the corresponding X-ray geometry, lead to very stable aggregates. In particular, for CH⋯N and π⋯π dimers (Figure 6a and Figure 6c, respectively), interaction energies of 9.82 and 11.25 kcal/mol have been computed. TDDFT calculations on these aggregates (Tables S3–S7) lead, obviously, to two-fold (S_1_^(1)^, S_1_^(2)^) and four-fold (S_1_^(1)^, S_1_^(4)^) splitting of the original ‘monomeric’ S_1_ for the dimers and the tetramers, respectively. In the case of the CH⋯N aggregates (Figure 6a,b), the lower S_1_^(1)^ state gradually redshifts (by 0.06 eV from monomer to dimer and then by 0.01 eV from dimer to tetramer), displaying an impressive increase in its oscillator strength, from 0.25 (monomer) to 0.74 (dimer) and then 1.64 (tetramer). The π⋯π stacked dimeric and tetrameric species (Figure 6c,d) show a similar S_1_^(1)^ redshift (by 0.07 and then 0.03 eV, respectively, with respect to the monomer), though the stronger split level displays a lesser increase in oscillator strength (*f* = 0.36 for S_1_^(2)^ in the dimer and 0.47 for S_1_^(4)^ in the tetramer). For the ‘combined’ CH⋯N/π⋯π tetrameric aggregate (Figure 6e), S_1_^(1)^ redshifts by 0.13 eV with respect to the monomer and the larger S_1_^(i)^ oscillator strengths are *f* = 0.34 (S_1_^(2)^) and *f* = 0.81 (S_1_^(4)^). The computed shift well approximates the separation between HEF and LEF excitations, 0.16 eV, observed in crystals. Calculations, therefore, seem to explain the presence of the two fluorescences as the result of both the supramolecular interactions and the strong intensification of the ‘aggregate’ S_1_ level, the latter mainly due to the strong HB characterizing the **TT-CCH** crystal structure.

## 3. Discussion

**Figure 7:**
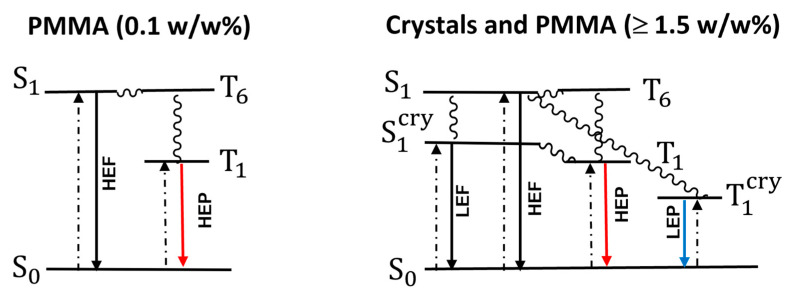
Simplified Jablonski diagrams.

The origin of the complex, multicomponent, and excitation-dependent photoluminescent behaviour of **TT-CCH** can be interpreted through combined optical, structural, and computational investigations. The sharp increase in quantum yield from DCM (5 × 10^−5^ M) solutions (Φ = 2%) and PMMA blended films (Φ from <0.1% for the 0.1 *w*/*w*% film to 3% for the 5 *w*/*w*% film) to powders (16%) indicates a beneficial effect of intermolecular interactions on both fluorescence and phosphorescence. Reasonably, both CH⋯N and π⋯π stacking interactions contribute to rigidifying the molecules and inhibiting non-radiative deactivation channels, resulting in crystallization-enhanced emissive features. The behaviour detected in the solution is biased by the compound’s low solubility and the necessary use of relatively high concentrations (5 × 10^−5^ M) due to the very low molecular quantum efficiency. A better interpretation of the molecular emissive behaviour can be obtained through the investigation of blended PMMA films with low dye loading (0.1 *w*/*w*%). In this condition, the compound displays HEF and HEP, which are, therefore, reasonably interpreted as molecular emissive components. HEP appears in the PL spectrum only when selectively activated at low energy being otherwise overwhelmed by stronger HEF, but it represents the only long-lived component in delayed spectra. The pathway leading to HEP has been disclosed through computational studies suggesting an easy ISC from S_1_ to an almost isoenergetic T_6_ of partially different character, which then populates T_1_ through IC (see the simplified Jablonski diagram reported in Figure 7). By increasing the dye loading in the PMMA films, two additional emissions (LEF and LEP), whose origin is, therefore, of supramolecular nature, are observed in the PL spectrum at proper excitation. LEP can also be disclosed in Ph spectra taken at different delays, as shown in Figure 2. This multicomponent behaviour is magnified for powders where, at high excitation energy, HEF, LEF and HEP are simultaneously activated. In this case, the easy S_1_-T_6_ ISC, together with rigidification and protection from molecular oxygen quenching played by intermolecular interactions, results in quite an intense HEP. Moreover, the effect of manual grinding on crystals of **TT-CCH**, which results in the attenuation of LEF intensity, supports the different (molecular vs. supramolecular) origin of the high- and the low-energy fluorescences, respectively.

Such multicomponent emissive behaviour is not frequently observed for common organic dyes, but this is quite often so for members of the **TT** family. As already mentioned, **TT**s display in the solid state, among others, a low-energy, long-lived emission attributed to π-π stacking interactions stabilizing the triplet state. On the other hand, the fluorescence of aggregated origin has been previously observed only for 3-(9H-carbazol-9-yl)triimidazo[1,2-*a*:1′,2′-*c*:1″,2″-*e*][1,3,5]triazine, **TT-(N)-Cz** and assigned, as well, to π-π stacking interactions [44]. In the present case, computational studies on different kinds of small aggregates point to a synergy between CH⋯N and π-π interactions, resulting in a lowering of the first excited singlet and triplet states. More importantly, strong CH⋯N hydrogen bonds, as determined in the crystal structure of **TT-CCH**, are recognized as responsible for an impressive increase in the oscillator strength of the first excited singlet, thus explaining the appearance of LEF. Importantly, to our knowledge, the present case is one of the rare examples of multiple fluorescences associated with both molecular and H-bonded supramolecular species [42]. Moreover, **TT-CCH** represents, so far, the first example among **TTs** displaying strong CH⋯N hydrogen bonds thanks to the presence of the -C≡C-H donor group, opening the further investigation of this functional group in combination with stronger HB acceptors on the **TT** scaffold.

## 4. Materials and Methods

### 4.1. General Information

All reagents have been purchased from chemical suppliers and used without further purification unless otherwise stated. DBU was freshly distilled from CaH_2_. **TT-Br** and **TT-I** have been prepared according to the literature procedures [17,49].

Melting points were recorded on a hot-stage microscope (Reichert Thermovar, Utrecht, The Netherlands). Precoated silica gel PET foils (Merck KGaA, Darmstadt, Germany) were used for TLC analyses. GLC-MS analyses were recorded with an (Agilent Technologies, Santa Clara, CA, USA) 6890 N gas chromatograph interfaced with an (Agilent Technologies, Santa Clara, CA, USA) MS5973 mass detector using an (Agilent Technologies, Santa Clara, CA, USA) J&W DB-5ms (30 m × 0.25 mm × 0.25 μm) column. LC-MS analyses were recorded on a Thermo Fisher LCQ Fleet Ion Trap Mass Spectrometer equipped with UltiMate™ 3000 HPLC system or on an (Waters Corporation, Milford, CT, USA) Acquity UPLC instrument (Phase A 95/5 H_2_O/ACN + 0.1% Formic Acid, Phase B 5/95 H_2_O/ACN + 0.1% Formic Acid; Acquity UPLC 2.1 × 100 mm column, BEH C18, 1.7 μm; Flow 0.6 mL/min) coupled with an (Waters Corporation, Milford, CT, USA) Acquity QDa Water mass spectrometer (Probe temperature: 600 °C; ESI capillary voltage 1.5 V; Cone voltage 15 V; Mass range 60–1000). HRMS spectra were obtained using Synapt G2-S*i* QTof mass spectrometer with Zspray^TM^ ESI-probe for electrospray ionization (Waters Corporation, Milford, CT, USA). Purifications by flash chromatography were performed using Merck 60 silica gel (Merck KGaA, Darmstadt, Germany).

^1^H-NMR and ^13^C-NMR spectra were recorded with a Bruker AVANCE-400 instrument (Bruker Italia Srl, Milano, Italy) or a Jeol 500 spectrometer (Jeol Italia SPA, Basiglio, Italy), referring chemical shifts to the residual solvent signal. The following notation was used in order to report NMR spectra: s = singlet, d = doublet, m = multiplet.

Films of **TT-CCH** dispersed in polymethylmethacrylate (PMMA) were prepared by spin coating (2000 rpm, 60 s) a dichloromethane solution **TT-CCH** = 0.1, 1.5, 3 or 5 wt%; PMMA = 10 wt% (with respect to the solvent) on a quartz substrate.

### 4.2. Synthesis of 3-Ethynyltriimidazo[1,2-*a*:1′,2′-*c*:1″,2″-*e*][1,3,5]triazine (TT-CCH)

**TT-CCH** has been prepared according to two different synthetic routes (Scheme 1) involving Sonogashira reaction of either **TT-Br** with (triisopropylsilyl)acetylene followed by desilylation with TBAF (Route 1) or **TT-I** with 2-methylbut-3-yn-2-ol and further deprotection under basic conditions (Route 2) [45]. All compounds have been characterized by ^1^H and ^13^C NMR, mass spectroscopy and HRMS. The synthesis and characterization of **TT-CCH** and the two intermediates, namely **TT-CC-TIPS** and **TT-CC-*^i^*PrOH,** have never been reported before.

#### 4.2.1. Route 1

Step 1—Synthesis of 3-((triisopropylsilyl)ethynyl)triimidazo[1,2-*a*:1′,2′-*c*:1″,2″-*e*][1,3,5]triazine (**TT-CC-TIPS**): **TT-Br** (554 mg, 2 mmol), Pd(MeCN)_2_Cl_2_ (21 mg, 0.08 mmol), Xantphos (28 mg, 0.048 mmol) and CuI (7.6 mg, 0.04 mmol) were introduced into a 25 mL two-neck flask equipped with a condenser and magnetic stirring. Three vacuum/argon cycles were carried out, and anhydrous DMF (9 mL), DBU (1 mL) and (triisopropylsilyl)acetylene (TIPS-acetylene) (0.67 mL) were added, in this order, through a silicon septum. The system was heated at 90 °C in an oil bath for 4 h. The resulting mixture, once at room temperature, was added to 70 mL of NH_4_Cl(sat), and the aqueous phase was extracted with EtOAc (100 mL × 3). The combined organic phases were concentrated under vacuum. Purification was performed by flash chromatography on silica gel using a mixture of petroleum ether/EtOAc (45/55). **TT-CC-TIPS** (625 mg, 83% yield) was recovered as a yellow solid with a melting point of 120–122 °C.

^1^H NMR (500 MHz, CDCl_3_, TMS, 298 K, ppm): δ 7.78 (d, *J* = 1.7 Hz, 1H), 7.77 (d, *J* = 1.7 Hz, 1H), 7.44 (s, 1H), 7.27–7.25 (m, 2H), 1.20–1.17 (m, 21H).

^13^C NMR (125 MHz, CDCl_3_, TMS, 298 K, ppm): δ 135.47, 134.89, 134.35, 134.22, 129.47, 129.18, 111.54, 111.08, 102.21, 91.86, 18.61, 11.42.

ESI-MS *m*/*z* 379 [M + H]^+^.

HRMS (ESI/Q-TOF) m/z: [M + Na]^+^ Calcd for C_20_H_26_N_6_NaSi 401.1886; Found 401.1883.

Step 2—Desilylation of **TT-CC-TIPS** to **TT-CCH**: **TT-CC-TIPS** (378 mg, 1 mmol) was introduced into a 25 mL two-neck flask equipped with magnetic stirring. Three vacuum/argon cycles were carried out, and anhydrous THF (12.5 mL) was added through a silicon septum. The system was cooled to 0 °C with an ice bath, and then TBAF 1 M in THF was slowly added (1.5 mL). The resulting mixture was allowed to return to room temperature by removing the cold bath and stirring for 2 h. The crude was added to 50 mL of NaHCO_3_(sat), and the aqueous phase was extracted with DCM (100 mL × 3). The combined organic phases were concentrated under vacuum, and purification was performed by flash chromatography on silica gel using a mixture of EtOAc and petroleum ether (90:10). **TT-CCH** (202 mg, 91% yield) was recovered as a white solid with a melting point >350 °C. Crystals suitable for X-ray diffraction analysis were obtained by slow evaporation from a DCM/MeOH solution.

^1^H NMR (500 MHz, CDCl_3_, TMS, 298 K, ppm): δ 7.82 (d, *J* = 1.7 Hz, 1H), 7.80 (d, *J* = 1.7 Hz, 1H), 7.52 (d, *J* = 0.6 Hz, 1H), 7.39 (d, *J* = 1.7 Hz, 1H), 7.29 (d, *J* = 1.7 Hz, 1H), 3.80 (s, 1H).

^13^C NMR (125 MHz, CDCl_3_, TMS, 298 K, ppm): δ 135.58, 135.25, 134.85, 134.68, 129.58, 129.41, 111.68, 111.35, 109.58, 86.64, 70.48.

ESI-MS: *m*/*z* 223 [M + H]^+^.

HRMS (ESI/Q-TOF) *m*/*z*: [M + H]^+^ Calcd for C_11_H_7_N_6_ 223.0732; Found 223.0734.

#### 4.2.2. Route 2

Step 1—Synthesis of 2-methyl-4-(triimidazo[1,2-*a*:1′,2′-*c*:1″,2″-*e*][1,3,5]triazin-3-yl)but-3-yn-2-ol (**TT-CC-*^i^*PrOH**): **TT-I** (0.300 g; 0.926 mmol), Pd(PPh_3_)_2_Cl_2_ (0.032 g, 0.046 mmol), dry NEt_3_ (15 mL) and dry DMF(7.5 mL) were introduced into a 100 mL dry Schlenk flask equipped with a magnetic stirrer under Ar atmosphere. To this mixture, 2-methylbut-3-yn-2-ol (0.180 mL; 1.85 mmol) and CuI (0.016 g; 0.083 mmol) were further added, and the system was heated under static Ar atmosphere at 110 °C for 4 h. The resulting brown reaction mixture was then cooled to room temperature and evaporated to dryness. The crude reaction mixture was purified by gravimetric chromatography on SiO_2_ with DCM/MeOH as eluents (R_f_ = 0.35 in DCM/MeOH = 94/6) to give **TT-CC-*^i^*PrOH** as a white solid (0.180 g, 0.642 mmol, yield: 69%).

^1^H NMR (400 MHz, CD_3_CN, 298 K, ppm): δ 7.86 (d, *J* = 1.61 Hz, 1H), 7.82 (d, *J* = 1.63 Hz, 1H), 7.40 (s, 1H), 7.31 (d, *J* = 1.63 Hz, 1H), 7,28 (d, *J* = 1.61 Hz, 1H), 3.75 (s, 1H), δ 1.62 (s, 6H).

^13^C NMR (101 MHz, CD_3_CN, 298 K, ppm) δ 136.92, 136.52, 134.29, 129.89, 129.67, 112.39, 112.16, 110.64, 103.59, 69.76, 65.88, 31.36, 31.27.

ESI-MS: *m*/*z* 263 [M]^+^-H_2_O.

HRMS (ESI/Q-TOF) *m*/*z*: [M + Na]^+^ Calcd for C_14_H_12_N_6_Ona 303.0970; Found 303.0970.

Step 2—Hydrolysis of **TT-CC-*^i^*PrOH** to **TT-CCH**: **TT-CC-*^i^*PrOH** (0.236 g; 0.84 mmol), NaOH (0.155 g; 3.88 mmol) and anhydrous toluene (15 mL) were introduced into a 50 mL Schlenk flask equipped with magnetic stirring. Three vacuum/nitrogen cycles were carried out, and the system was heated at 110 °C for 10 h. The reaction is then cooled to room temperature, filtered on a Buchner, and the solvent removed in vacuum. The crude reaction mixture is purified by flash chromatography on SiO_2_ with DCM/CH_3_CN as eluents (R_f_ = 0.48 in DCM/CH_3_CN =1/1) to give **TT-CCH** as a white solid (0.090 g, 0.40 mmol, yield: 48%). The purity of **TT-CCH** obtained by this route was comparable to that obtained by route 1 as confirmed by ^1^H NMR analysis.

### 4.3. Single-Crystal X-ray Studies

X-ray data of **TT-CCH** have been collected on a Rigaku XtaLAB Synergy S X-ray diffractometer equipped with a CCD HyPix 6000 detector (Rigaku Co., Tokyo, Japan) operated with a mirror-monochromated microfocus Cu-Kα radiation (λ = 1.54184 Å) at 50 kV and 1.0 mA. The structure has been solved using direct methods and refined with SHELXL-18 [54] using a full-matrix least-squares procedure based on F^2^ using all data. Hydrogen atoms have been placed at geometrically estimated positions. Details relating to the crystal and the structural refinement are presented in Table S1. Full details of crystal data and structure refinement, in CIF format, are available as Supplementary Information. CCDC reference number: 2332846.

### 4.4. Computational Details

DFT and TDDFT calculations on isolated ‘gas-phase‘ **TT-CCH** and on its dimers and tetramers were performed with Gaussian 16 program (Revision A.03) [55] using the 6-311++G(d,p) basis set. Their geometries have been optimized starting from the corresponding X-ray ones. Based on previous theoretical results as obtained on the parent cyclic triimidazole and its derivatives [17,18,44,46,47,48,49,50,51,52,53], we adopted the ωB97X [56] functional for DFT calculations. This function was, in fact, demonstrated to be suitable to correctly treat at the same time not only ground and excited states properties but also intermolecular interactions, in particular π-π interactions, which play a key role in the photophysics of **TT** and its derivatives. In fact, the PBE0 and CAM-B3LYP functionals were previously found to accurately reproduce the absorption spectrum of the isolated monomers but failed to provide stable π-π stacked dimers. On the other hand, the B97D functional, while providing stable π-π dimers, was found to be unstable for TDDFT calculations.

### 4.5. Photophysical Characterization

Photoluminescence quantum yields have been measured using a C11347 Quantaurus–Absolute Photoluminescence Quantum Yield Spectrometer, equipped with a 150 W Xenon lamp, an integrating sphere and a multichannel detector (Hamamatsu Photonics K.K, Shizuoka, Japan). Steady-state emission and excitation spectra and photoluminescence lifetimes have been obtained using an FLS 980 (Edinburg Instrument Ltd., Livingston, UK) spectrofluorimeter. The steady-state measurements have been recorded by a 450 W Xenon arc lamp. Photoluminescence lifetime measurements have been performed using an EPLED-300 (Edinburg Instrument Ltd., Livingston, UK) and microsecond flash Xe-lamp (60 W, 0.1 ÷ 100 Hz) with data acquisition devices time-correlated single-photon counting (TCSPC) and multi-channel scaling (MCS) methods, respectively. Average lifetimes are obtained as τ_av_ = $\frac{\sum A_{i}\tau_{i\;}^{2}}{\sum A_{i}\tau_{i}}$ from bi-exponential or three-exponential fits. Low-temperature measurements have been performed by immersion of the sample in a liquid N_2_ quartz dewar.

Delayed spectra have been collected with a NanoLog spectrofluorimeter composed of an iH320 spectrograph equipped with a PPD-850 single-photon detector module with Time-Gated Separation by exciting with a pulsed Xe lamp (Horiba Scientific, Piscataway, NJ, USA). The spectra are corrected for the instrument response.

## Figures and Tables

**Table 1:** Photophysical properties of **TT-CCH** in DCM (5 × 10^−5^ M), PMMA-blended film (5 *w*/*w*%) and crystals.

	298 K				77 K			Origin
	Φ%	λ_exc_ (nm)	λ_em_ (nm)	τ	λ_exc_ (nm)	λ _em_ (nm)	τ	
DCM	2	266, 287	363	n.d.				S_1_-S_0_
PMMA (5% *w*/*w*%)	3		342	0.99 ns				S_M_-S_0_
			383	1.46 ns				S_H_-S_0_
			439	0.16 ms				T_M_-S_0_
			522	6.9 ms				T_H_-S_0_
Crystals	16	299	314, 326, 338	n.d.	299	310, 327, 337	n.d.	S_M_-S_0_
		311, 320, 333	354, 367, 377	1.05 ns	311, 320, 333	354, 364, 383	1.47 ns	S_H_-S_0_
		370, 390	396, 420	0.25 ms	345, 370, 390	395, 419	6.39 ms	T_M_-S_0_
		395, 415, 450	545	4.66 ms	398, 422, 447	489, 523	44.84 ms	T_H_-S_0_

## Data Availability

The data presented in this study are available in article and Supplementary Materials.

## Supplementary Materials

The following supporting information can be downloaded at: https://www.mdpi.com/article/10.3390/molecules29091967/s1, Figures S1–S8: ^1^H NMR, ^13^C NMR and GC-MS spectra; Figures S9–S21: photophysical data; Figure S22: simulated absorption spectrum; Figure S23: electronic levels; Figure S24: molecular orbitals; Table S1: crystal data; Tables S2–S7: computational data.
