# Supplementary material for: 3-Ethynyltriimidazo[1,2-a:1′,2′-c:1″,2″-e][1,3,5]triazine Dual Short- and Long-Lived Emissions with Crystallization-Enhanced Feature: Role of Hydrogen Bonds and π-π Interactions
Source: Molecules. 2024 Apr 25;29(9):1967. doi: 10.3390/molecules29091967 (PMC11085060; doi:10.3390/molecules29091967)

checkCIF/PLATON report

Structure factors have been supplied for datablock(s) TT-CCH

THIS REPORT IS FOR GUIDANCE ONLY. IF USED AS PART OF A REVIEW PROCEDURE FOR PUBLICATION, IT SHOULD NOT REPLACE THE EXPERTISE OF AN EXPERIENCED CRYSTALLOGRAPHIC REFEREE.

No syntax errors found. CIF dictionary Interpreting this report

Datablock: TT-CCH

```
Bond precision:   C-C = 0.0019 A          Wavelength=1.54184

Cell:           a=13.35072(15)    b=4.63511(4)        c=16.41198(19)
                alpha=90          beta=96.7803(11)    gamma=90
Temperature:    296 K

                 Calculated                  Reported
Volume           1008.504(19)                1008.504(19)
Space group      P 21/c                      P 21/c
Hall group       -P 2ybc                     -P 2ybc
Moiety formula   C11 H6 N6                   C11 H6 N6
Sum formula      C11 H6 N6                   C11 H6 N6
Mr               222.22                      222.22
Dx,g cm-3        1.464                       1.464
Z                4                           4
Mu (mm-1)        0.805                       0.805
F000             456.0                       456.0
F000'            457.38
h,k,lmax         17,5,20                     17,5,20
Nref             2194                        2177
Tmin,Tmax        0.849,0.923                 0.595,1.000
Tmin'            0.773

Correction method= # Reported T Limits: Tmin=0.595 Tmax=1.000
AbsCorr = MULTI-SCAN

Data completeness= 0.992          Theta(max)= 80.142

R(reflections)= 0.0420( 2010)                         wR2(reflections)=
                                                      0.1164( 2177)

S = 1.050                   Npar= 154
```

The following ALERTS were generated. Each ALERT has the format
test-name_ALERT_alert-type_alert-level.
Click on the hyperlinks for more details of the test.

Alert level C

```
PLAT906_ALERT_3_C Large K Value in the Analysis of Variance ......      2.195 Check
```

Alert level G

```
PLAT142_ALERT_4_G s.u. on b - Axis Small or Missing .............    0.00004 Ang.
PLAT371_ALERT_2_G Long   C(sp2)-C(sp1) Bond  C2       - C10      .       1.41 Ang.
PLAT432_ALERT_2_G Short Inter X...Y Contact  C11      ..C11      .       3.20 Ang.
                                               2-x,2-y,1-z  =      3_776 Check
PLAT899_ALERT_4_G SHELXL2018 is Deprecated and Succeeded by SHELXL     2019/3 Note
PLAT899_ALERT_4_G SHELXL2018 is Deprecated and Succeeded by SHELXL     2019/3 Note
PLAT912_ALERT_4_G Missing # of FCF Reflections Above STh/L=  0.600         17 Note
PLAT969_ALERT_5_G The 'Henn et al.' R-Factor-gap value ..........       4.25 Note
              Predicted wR2: Based on SigI**2  2.74 or SHELX Weight 11.51
PLAT978_ALERT_2_G Number C-C Bonds with Positive Residual Density.          0 Info
PLAT992_ALERT_5_G Repd & Actual _reflns_number_gt Values Differ by          2 Check
```

0 **ALERT level A** = Most likely a serious problem - resolve or explain
0 **ALERT level B** = A potentially serious problem, consider carefully
1 **ALERT level C** = Check. Ensure it is not caused by an omission or oversight
9 **ALERT level G** = General information/check it is not something unexpected

0 ALERT type 1 CIF construction/syntax error, inconsistent or missing data
3 ALERT type 2 Indicator that the structure model may be wrong or deficient
1 ALERT type 3 Indicator that the structure quality may be low
4 ALERT type 4 Improvement, methodology, query or suggestion
2 ALERT type 5 Informative message, check

It is advisable to attempt to resolve as many as possible of the alerts in all categories. Often the minor alerts point to easily fixed oversights, errors and omissions in your CIF or refinement strategy, so attention to these fine details can be worthwhile. In order to resolve some of the more serious problems it may be necessary to carry out additional measurements or structure refinements. However, the purpose of your study may justify the reported deviations and the more serious of these should normally be commented upon in the discussion or experimental section of a paper or in the "special_details" fields of the CIF. checkCIF was carefully designed to identify outliers and unusual parameters, but every test has its limitations and alerts that are not important in a particular case may appear. Conversely, the absence of alerts does not guarantee there are no aspects of the results needing attention. It is up to the individual to critically assess their own results and, if necessary, seek expert advice.

Publication of your CIF in IUCr journals

A basic structural check has been run on your CIF. These basic checks will be run on all CIFs submitted for publication in IUCr journals (*Acta Crystallographica*, *Journal of Applied Crystallography*, *Journal of Synchrotron Radiation*); however, if you intend to submit to *Acta Crystallographica Section C* or *E* or *IUCrData*, you should make sure that full publication checks are run on the final version of your CIF prior to submission.

Publication of your CIF in other journals

Please refer to the *Notes for Authors* of the relevant journal for any special instructions relating to CIF submission.

PLATON version of 06/01/2024; check.def file version of 05/01/2024

Datablock TT-CCH - ellipsoid plot

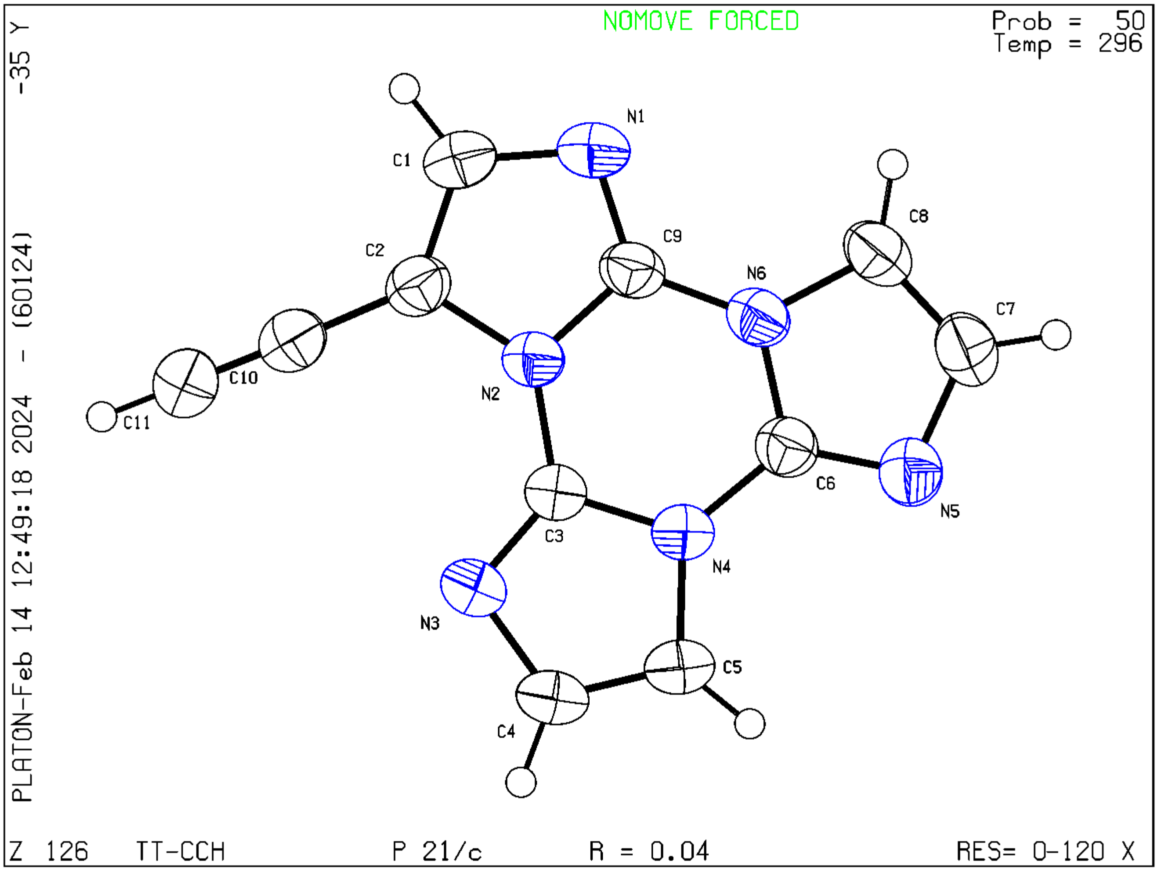

Supplement: Supplementary file 1 [file molecules-29-01967-s001.zip › checkcif.pdf]
