# Supplementary material for: 3-Ethynyltriimidazo[1,2-a:1′,2′-c:1″,2″-e][1,3,5]triazine Dual Short- and Long-Lived Emissions with Crystallization-Enhanced Feature: Role of Hydrogen Bonds and π-π Interactions
Source: Molecules. 2024 Apr 25;29(9):1967. doi: 10.3390/molecules29091967 (PMC11085060; doi:10.3390/molecules29091967)
Supplement: Supplementary file 1 [file molecules-29-01967-s001.zip › Supplementary Information.pdf]

3-Ethynyltriimidazo[1,2-*a*:1',2'-*c*:1'',2''-*e*][1,3,5]triazine Dual Short- and Long-Lived Emissions with Crystallization-Enhanced Feature: Role of Hydrogen Bonds and π-π Interactions

Daniele Malpicci[1,2], Daniele Maver[1,2], Elisabetta Rosadoni[3], Alessia Colombo[1,4], Elena Lucenti[2,4], Daniele Marinotto[2,4], Chiara Botta[5], Fabio Bellina[3], Elena Cariati[1,2,4,*] and Alessandra Forni[2,4,*]

Supplementary information

Table of Contents

1. NMR and GC-MS Spectra 2
2. Photophysical Data 10
3. Crystal data 17
4. Theoretical Studies..18

1. NMR and GC-MS Spectra

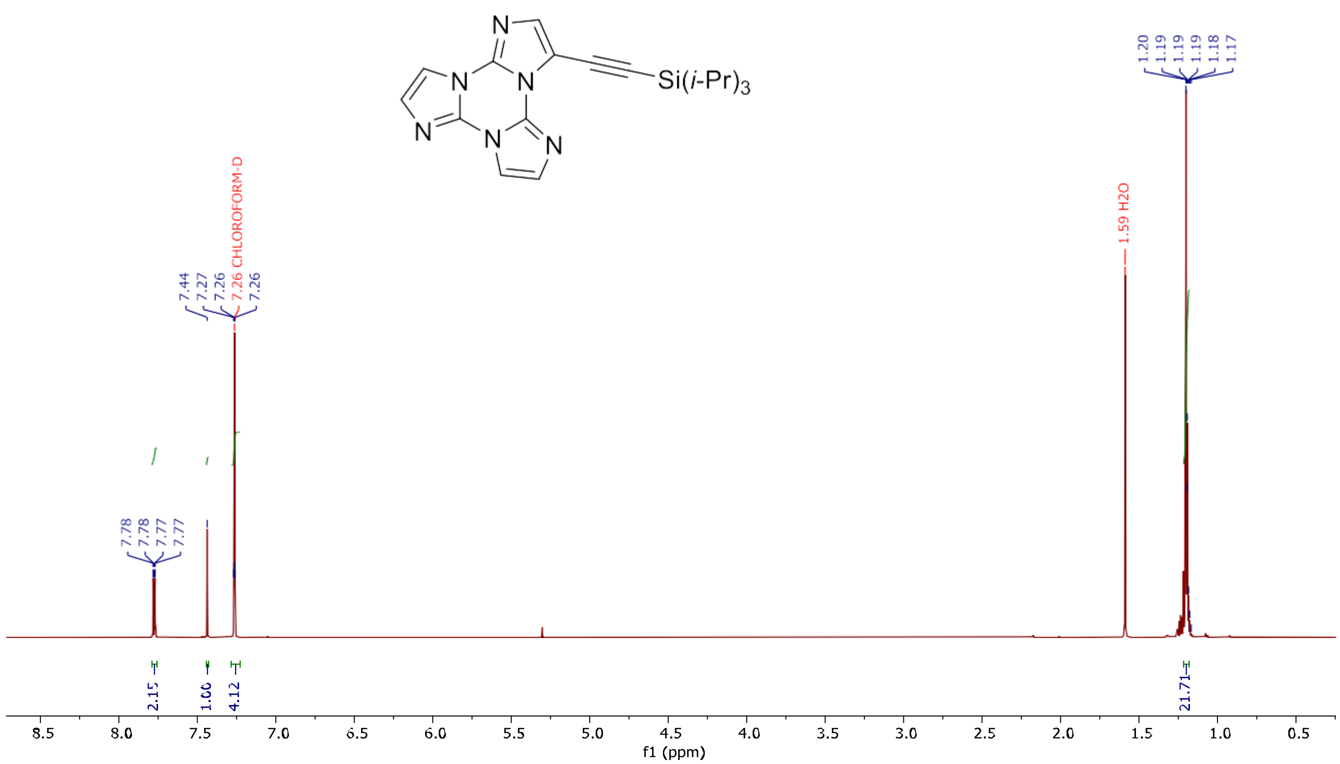

Figure S1. ^{1}H-NMR ($CDCl_3$, 500 MHz) of **TT-CC-TIPS**.

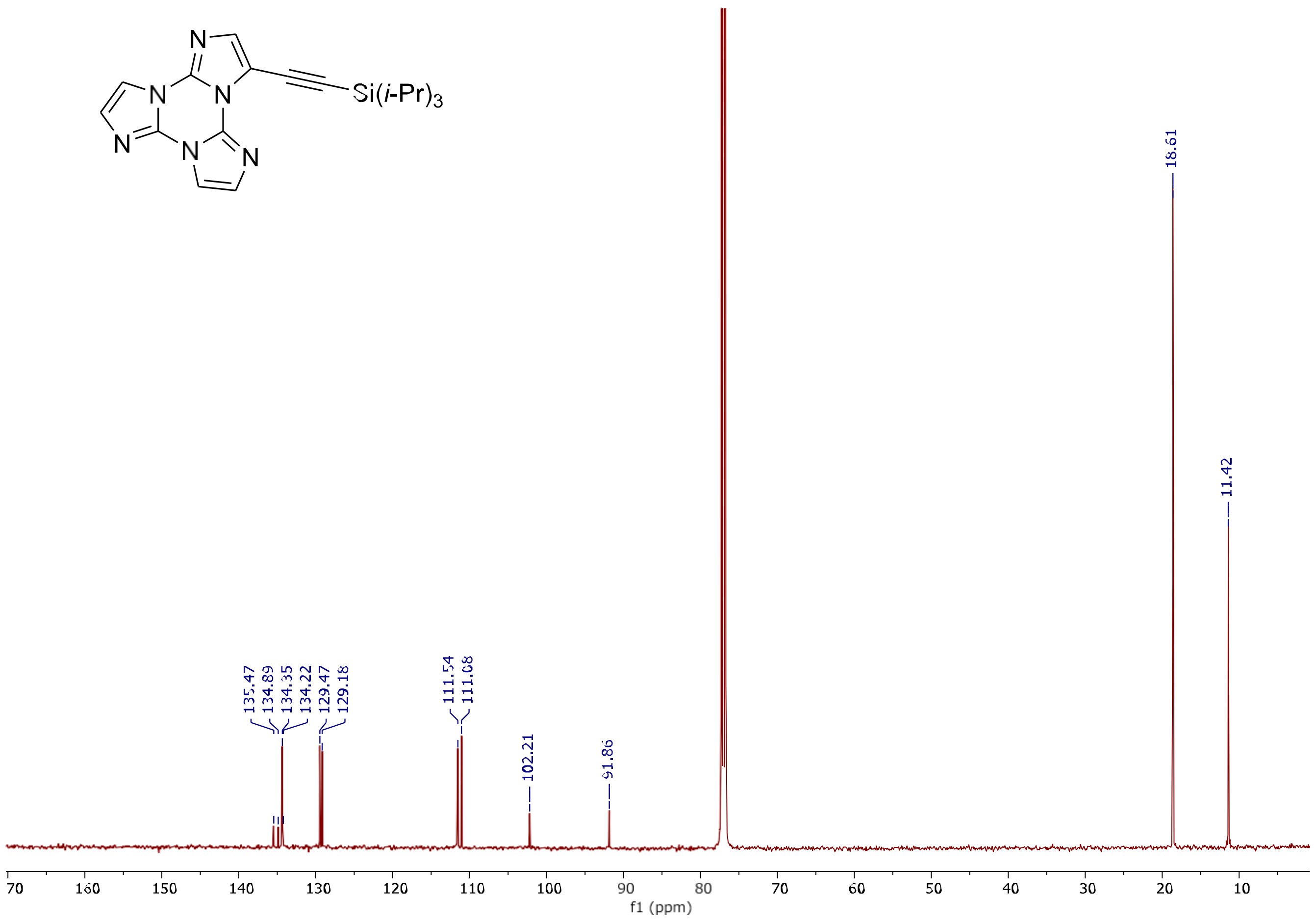

Figure S2. ^{13}C-NMR ($CDCl_3$, 125 MHz) of **TT-CC-TIPS**.

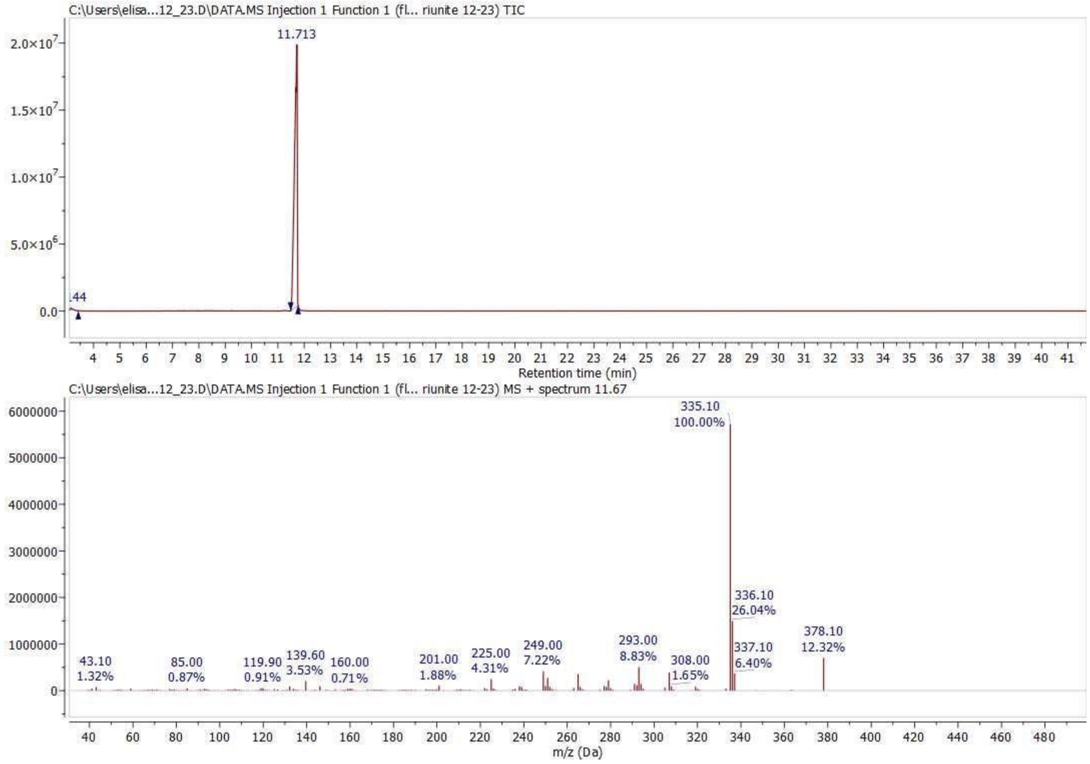

Figure S3. GC-MS(EI) profile of **TT-CCH**

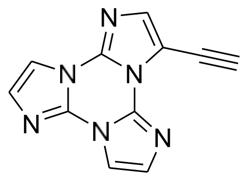

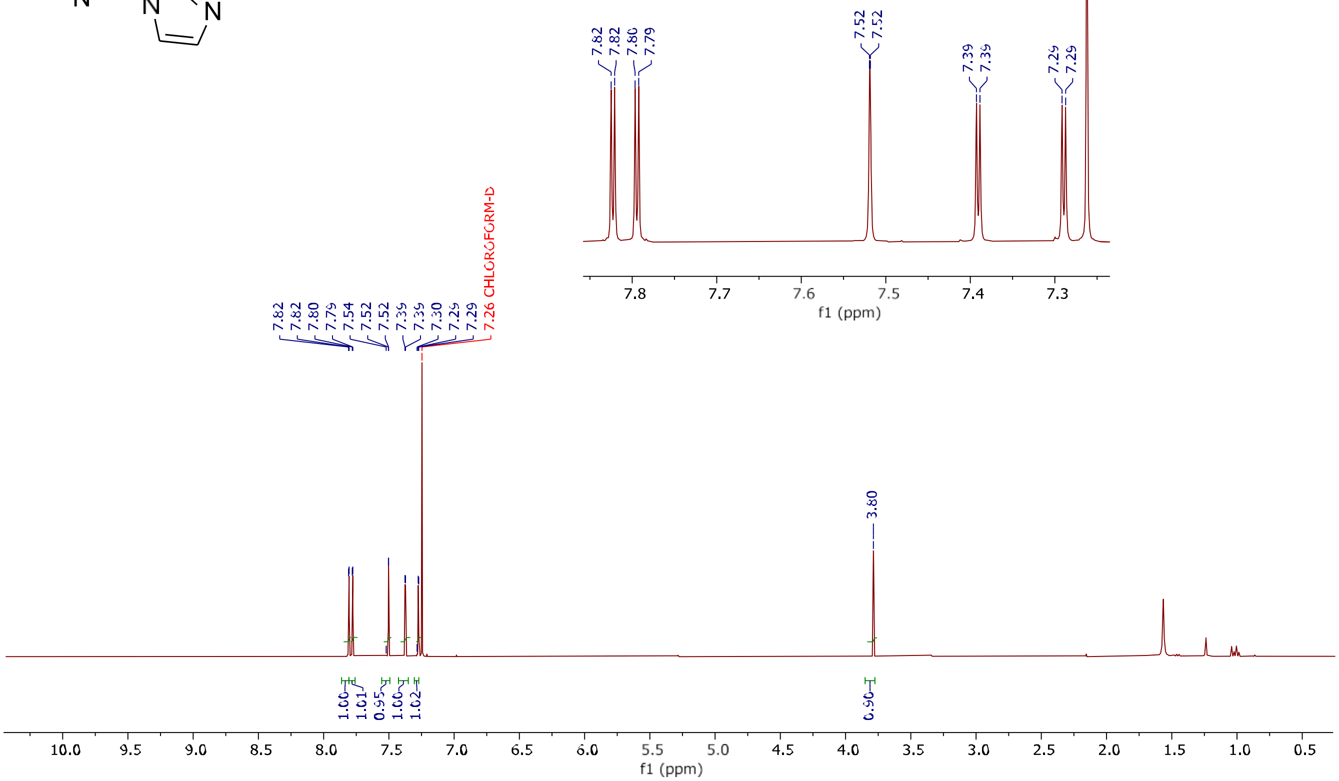

Figure S4. ^{1}H-NMR ($CDCl_3$, 500 MHz) of **TT-CCH**

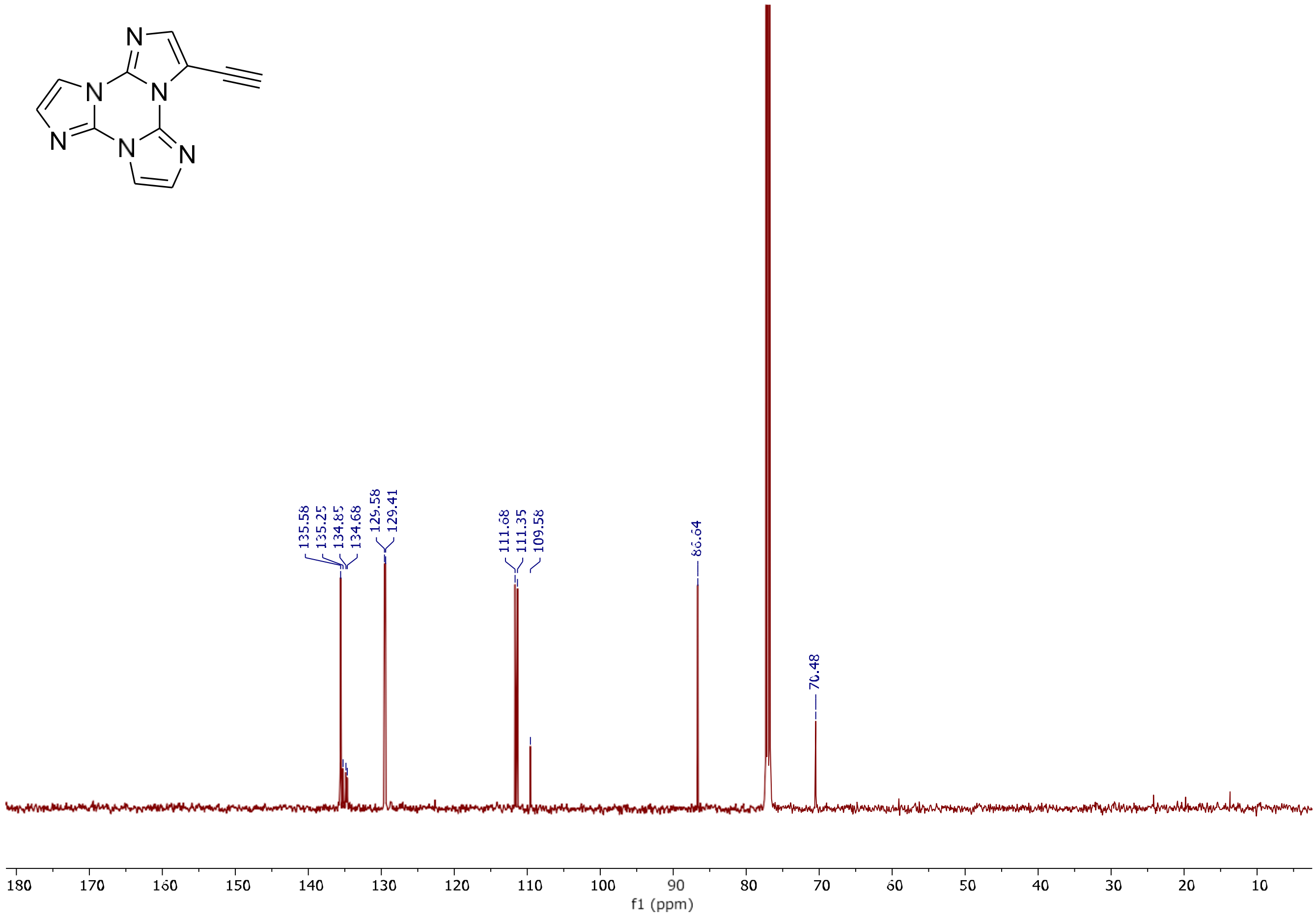

Figure S5. ^{13}C-NMR spectra ($CDCl_3$, 125 MHz) of **TT-CCH**

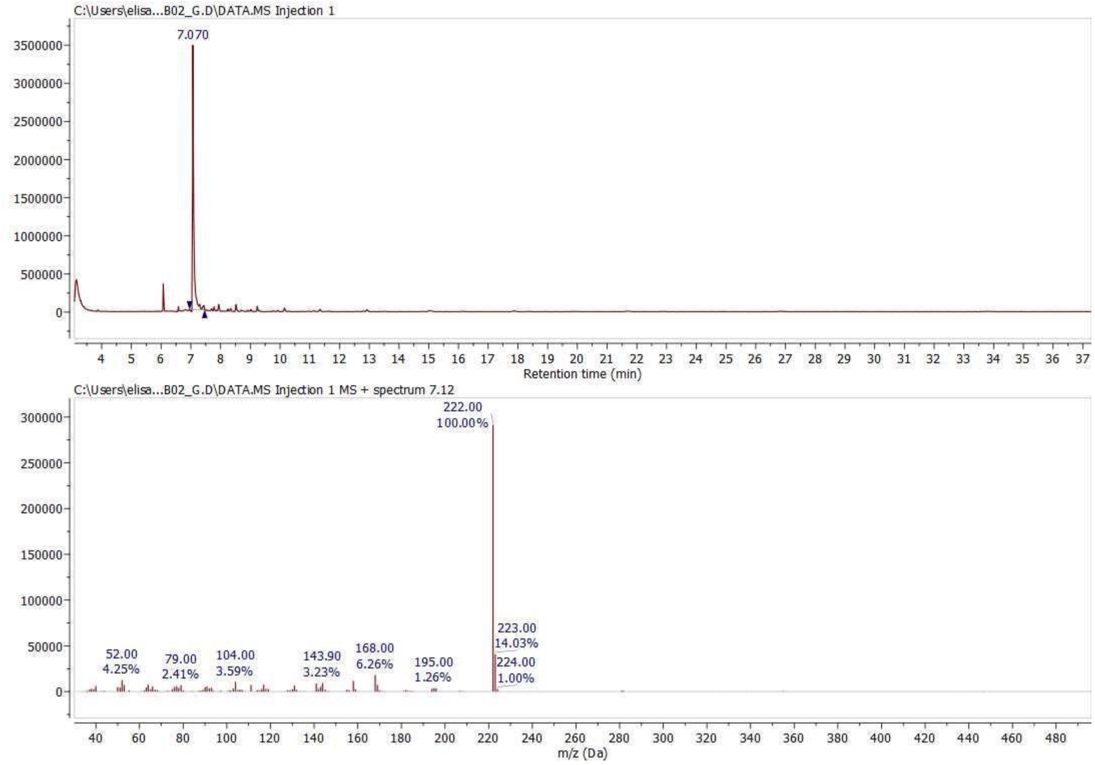

Figure S6. GC-MS(EI) profile of **TT-CCH**

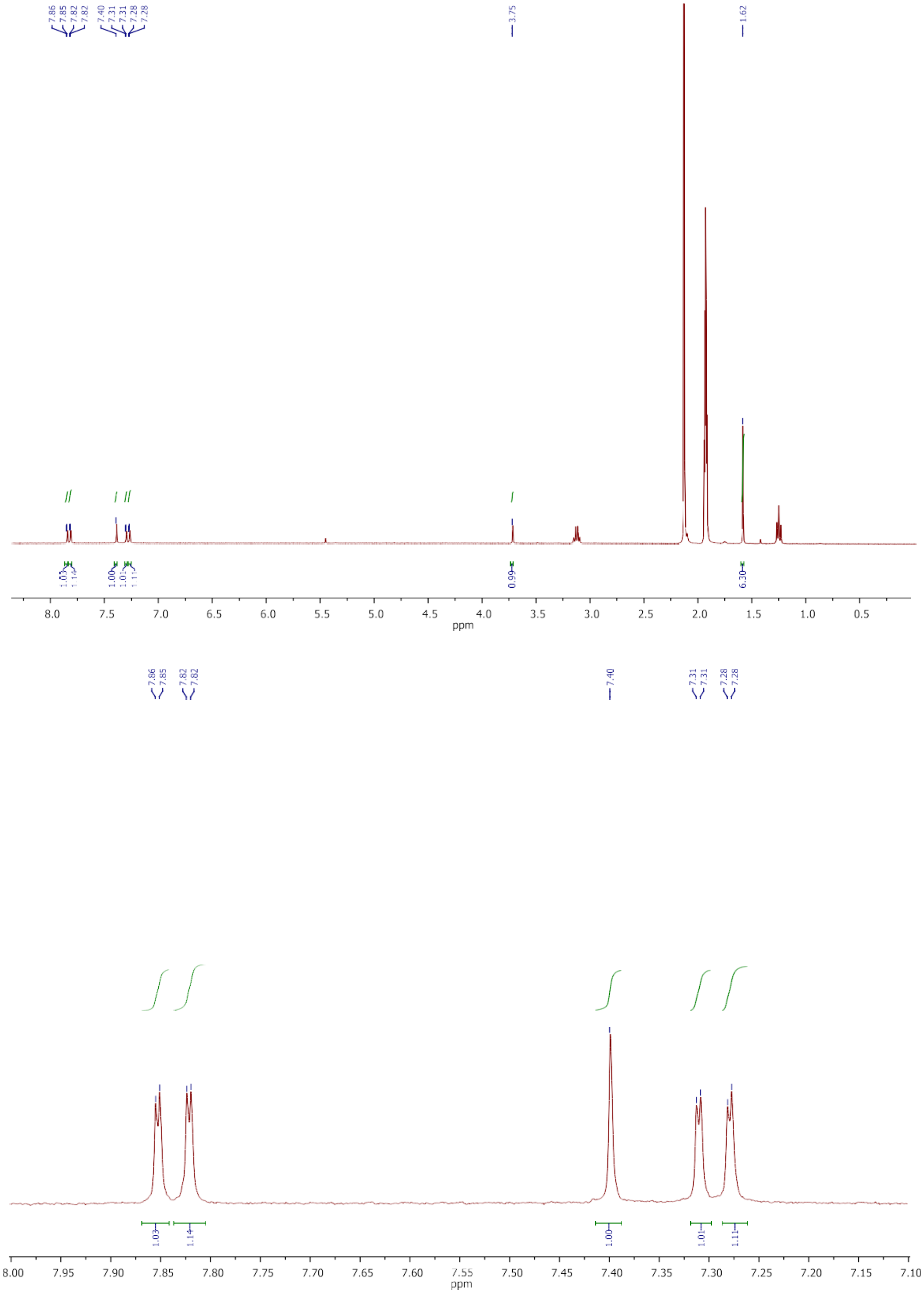

Figure S7. ^{1}H-NMR (CD_3CN, 400 MHz) of **TT-CC-^{i}PrOH** (top) with expansion of aromatic region (below).

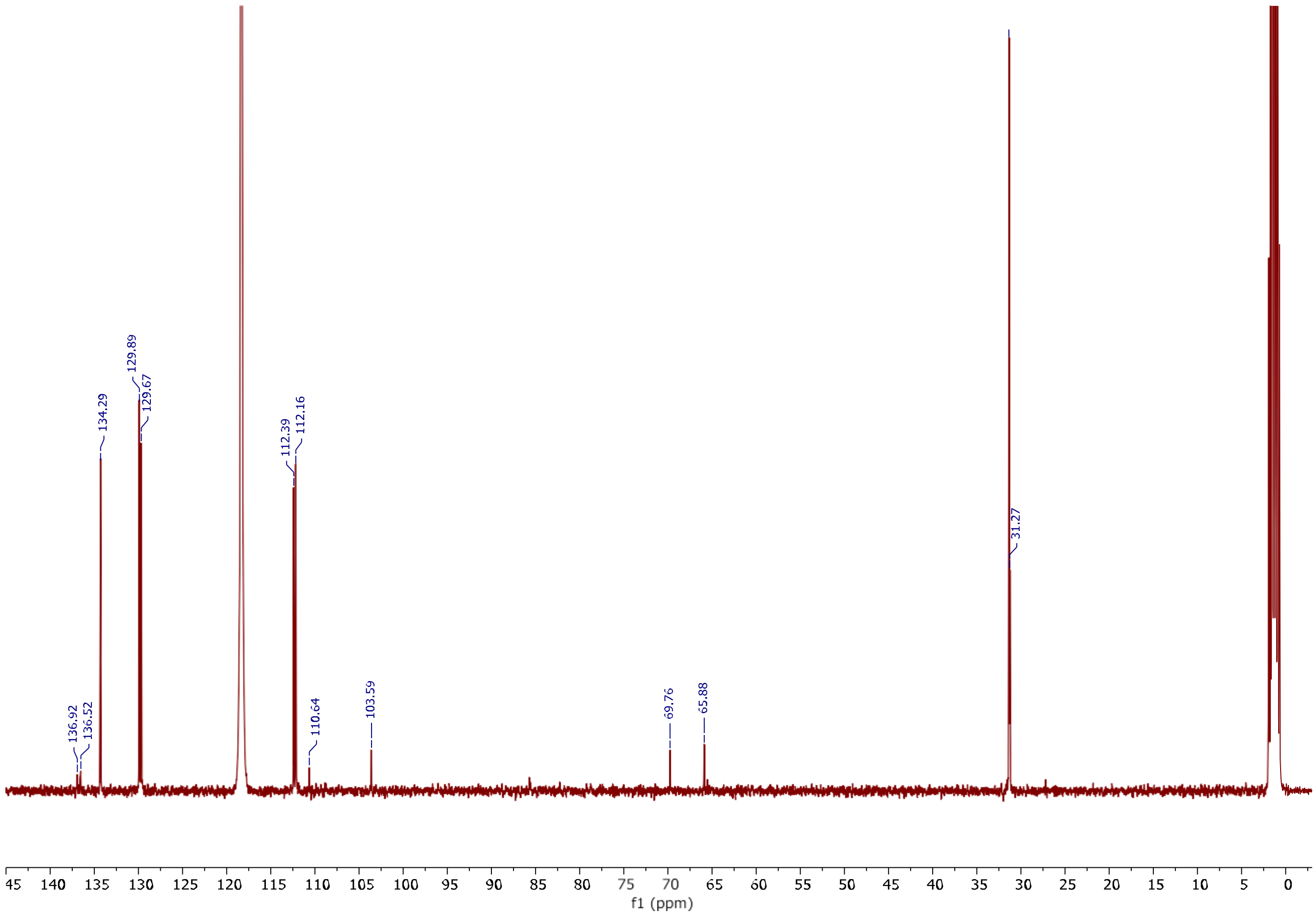

Figure S8. ^{13}C-NMR (CD_3CN, 101 MHz) of **TT-CC-*i*PrOH**.

2. Photophysical Data

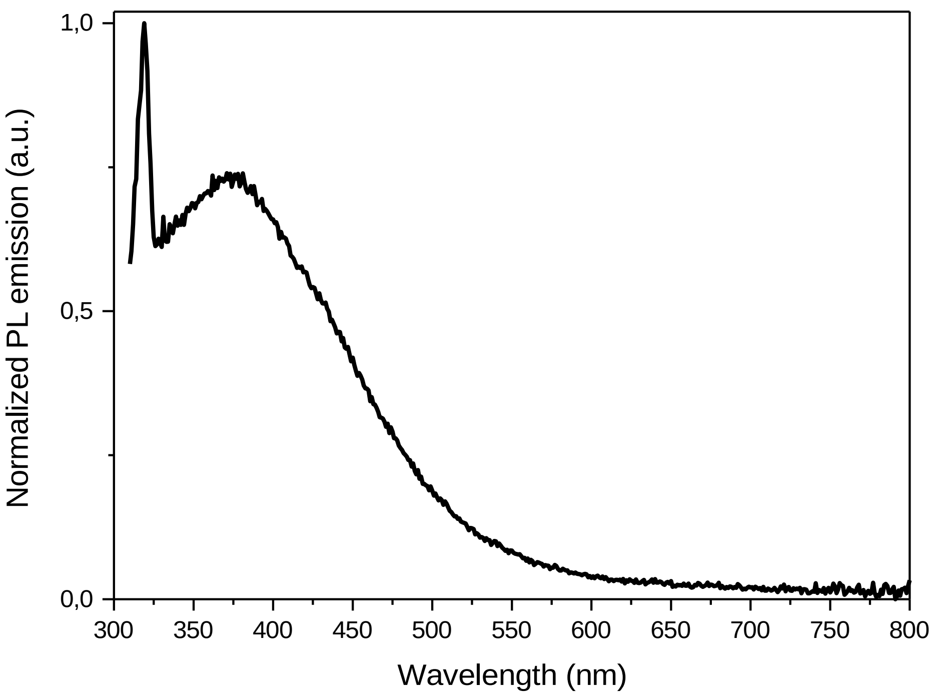

Figure S9. Normalized emission (λ_{exc} = 290 nm) of **TT-CCH** in DCM solution (1×10^{-5} M) at 298 K.

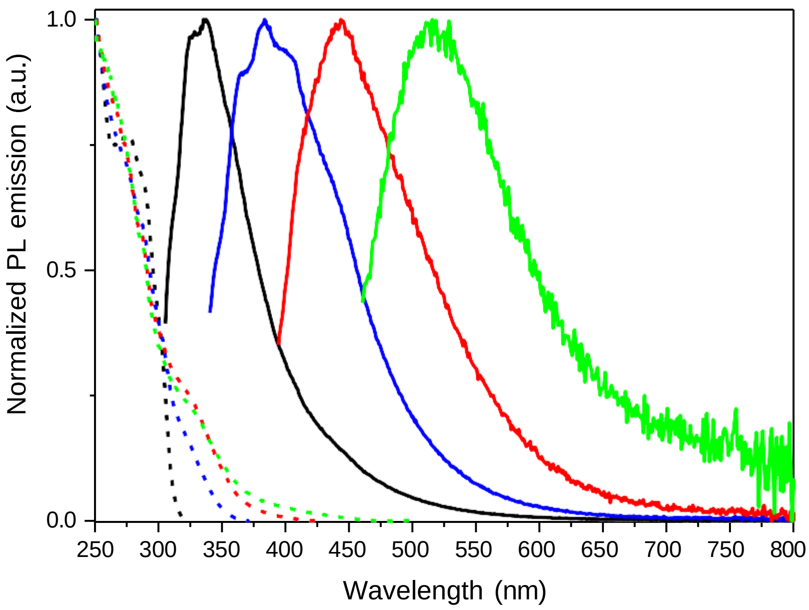

Figure S10. Normalized excitation (dashed lines: λ_{em} = 336 nm (black), 382 nm (blue), 442 nm (red) and 516 nm (green)) and normalized emission (continuous lines: λ_{exc} = 290 nm (black), 330 nm (blue), 380 nm (red) and 445 nm (green) of **TT-CCH** in PMMA film (1.5 wt%) at 298 K.

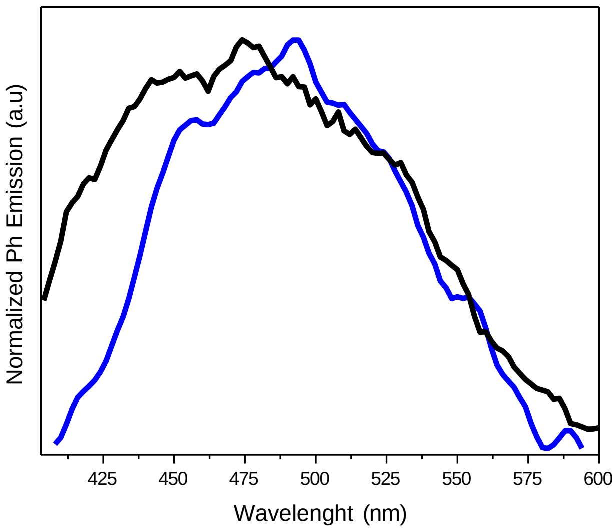

Figure S11. Normalized delayed spectra of **TT-CCH** in PMMA matrix (0.1 w/w%,) at short (40 µs delay, 200 µs window; black line) and long (4 ms delay, 20 ms window; blue line) delay times.

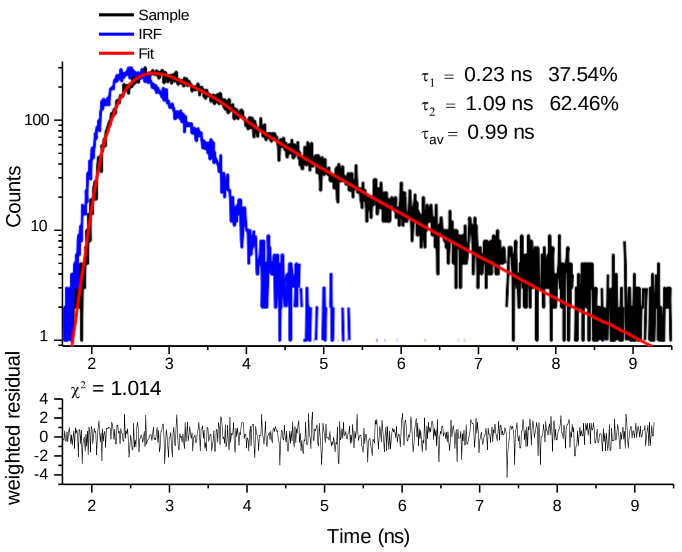

Figure S12. Lifetime measurement (λ_{exc} = 300 nm, λ_{em} = 336 nm) of **TT-CCH** in in PMMA film (5 wt%) at 298 K.

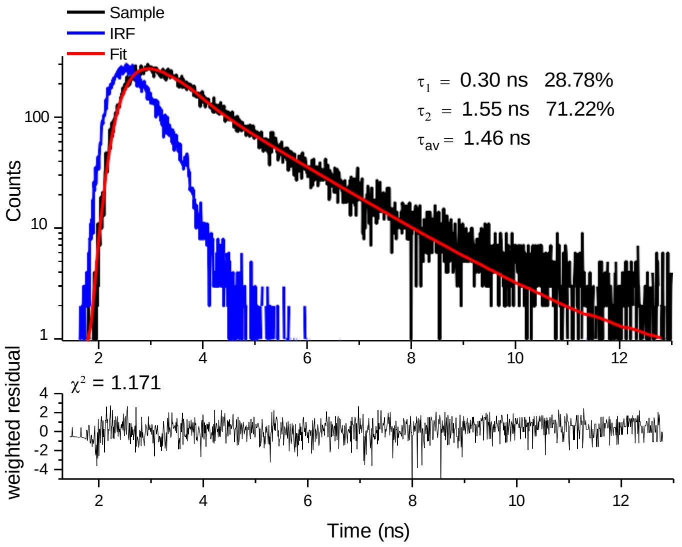

Figure S13. Lifetime measurement (λ_{exc} = 300 nm, λ_{em} = 383 nm) of **TT-CCH** in in PMMA film (5 wt%) at 298 K.

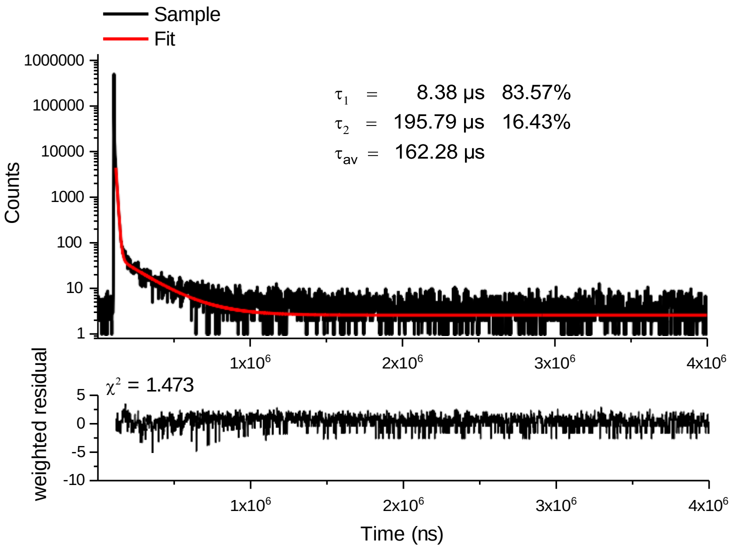

Figure S14. Lifetime measurement (λ_{exc} = 300 nm, λ_{em} = 445 nm) of **TT-CCH** in in PMMA film (5 wt%) at 298 K.

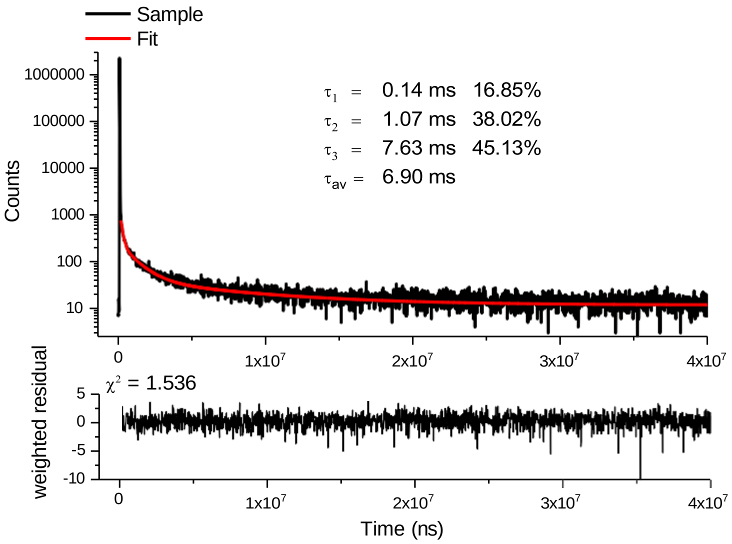

Figure S15. Lifetime measurement (λ_{exc} = 300 nm, λ_{em} = 516 nm) of **TT-CCH** in in PMMA film (5 wt%) at 298 K.

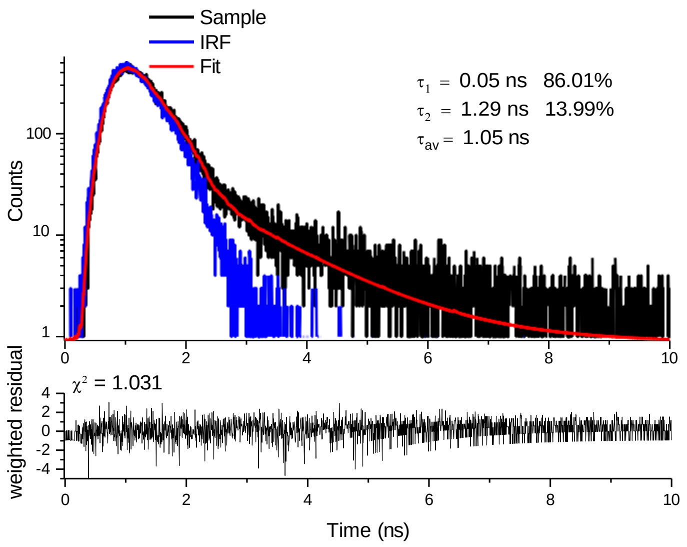

Figure S16. Lifetime measurement (λ_{exc} = 300 nm, λ_{em} = 370 nm) of **TT-CCH** crystals at 298 K.

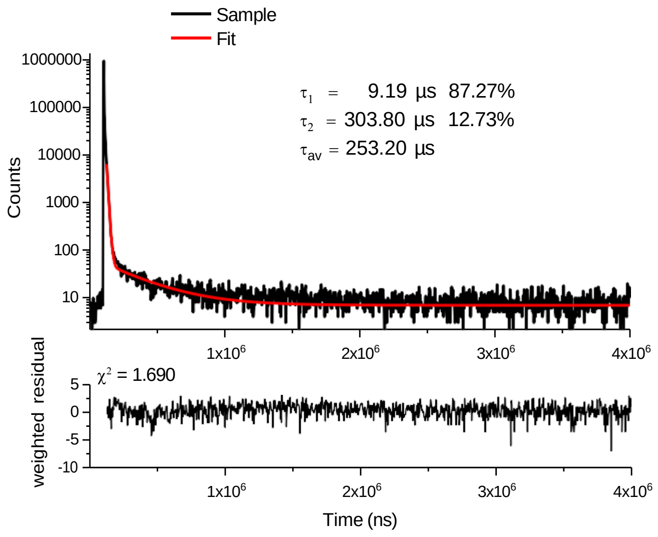

Figure S17. Lifetime measurement (λ_{exc} = 300 nm, λ_{em} = 418 nm) of **TT-CCH** crystals at 298 K.

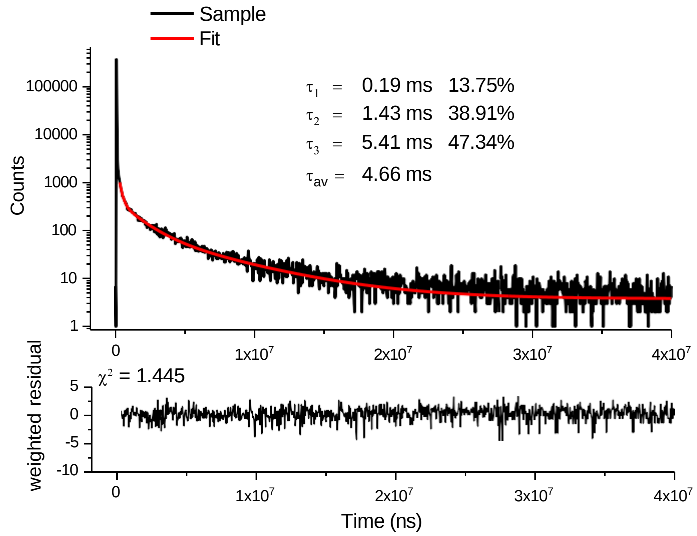

Figure S18. Lifetime measurement (λ_{exc} = 300 nm, λ_{em} = 520 nm) of **TT-CCH** crystals at 298 K.

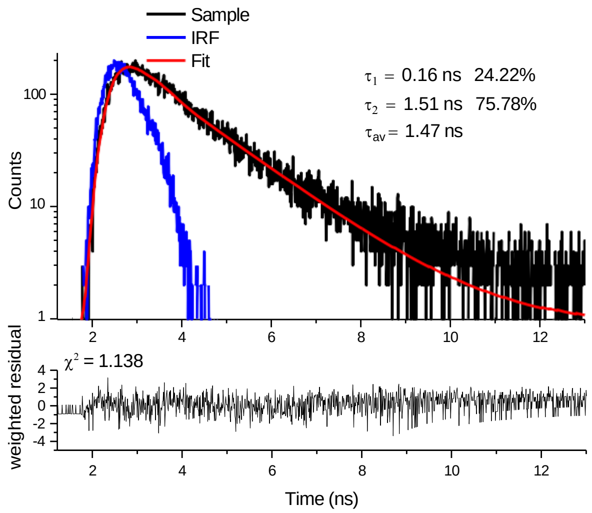

Figure S19. Lifetime measurement (λ_{exc} = 300 nm, λ_{em} = 379 nm) of **TT-CCH** crystals at 77 K.

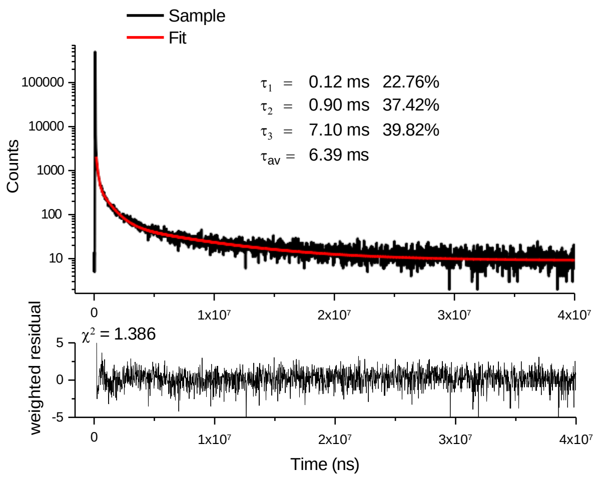

Figure S20. Lifetime measurement (λ_{exc} = 300 nm, λ_{em} = 419 nm) of **TT-CCH** crystals at 77 K.

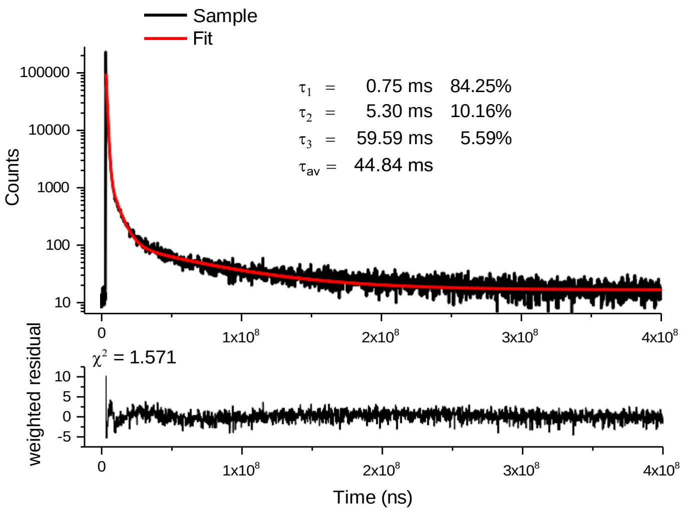

Figure S21. Lifetime measurement (λ_{exc} = 300 nm, λ_{em} = 525 nm) of **TT-CCH** crystals at 77 K.

3. Crystal data

Table S1 Crystal data, data collection and refinement details for **TT-CCH**

	**TT-CCH**
Crystal data	
Chemical formula	$C_{11}H_6N_6$
M_r	222.22
Crystal system	monoclinic
Space group	P 21/1 (No. 14)
Temperature [K]	296(2)
a [Å]	13.35072(15)
b [Å]	4.63511(4)
c [Å]	16.41198(19)
α [°]	90
β [°]	96.7803(11)
γ [°]	90
V [Å^3]	1008.504(19)
Z	4
μ(CuKα) [mm^{-1}]	0.805
Crystal size [mm]	0.32 × 0.17 × 0.10
Data collection	
No. of measured reflections	20327
No. of independent reflections	2177
No. of observed reflections, I > 2σ(I)	2010
R_{int}	0.044
R_{σ}	0.026
$(\sin \theta/\lambda)_{max}$ [Å^{-1}]	0.82
Refinement	
$R[F^2 > 2\sigma(F^2)]$	0.0440
$wR(F^2)$	0.1164
S	1.050
No. of reflections	2177
No. of parameters	154
No. of restraints	0
$\Delta\varrho_{max}$, $\Delta\varrho_{min}$ (e Å^{-3})	0.162, –0.235

4. Theoretical studies

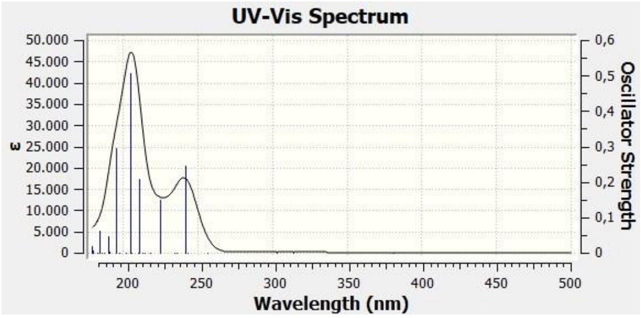

Figure S22. TD-ωB97X/6-311++G(d,p) absorption spectra of **TT-CCH** at the optimized geometry, obtained by convolution of the blue sticks with 0.2 eV of half-bandwidth.

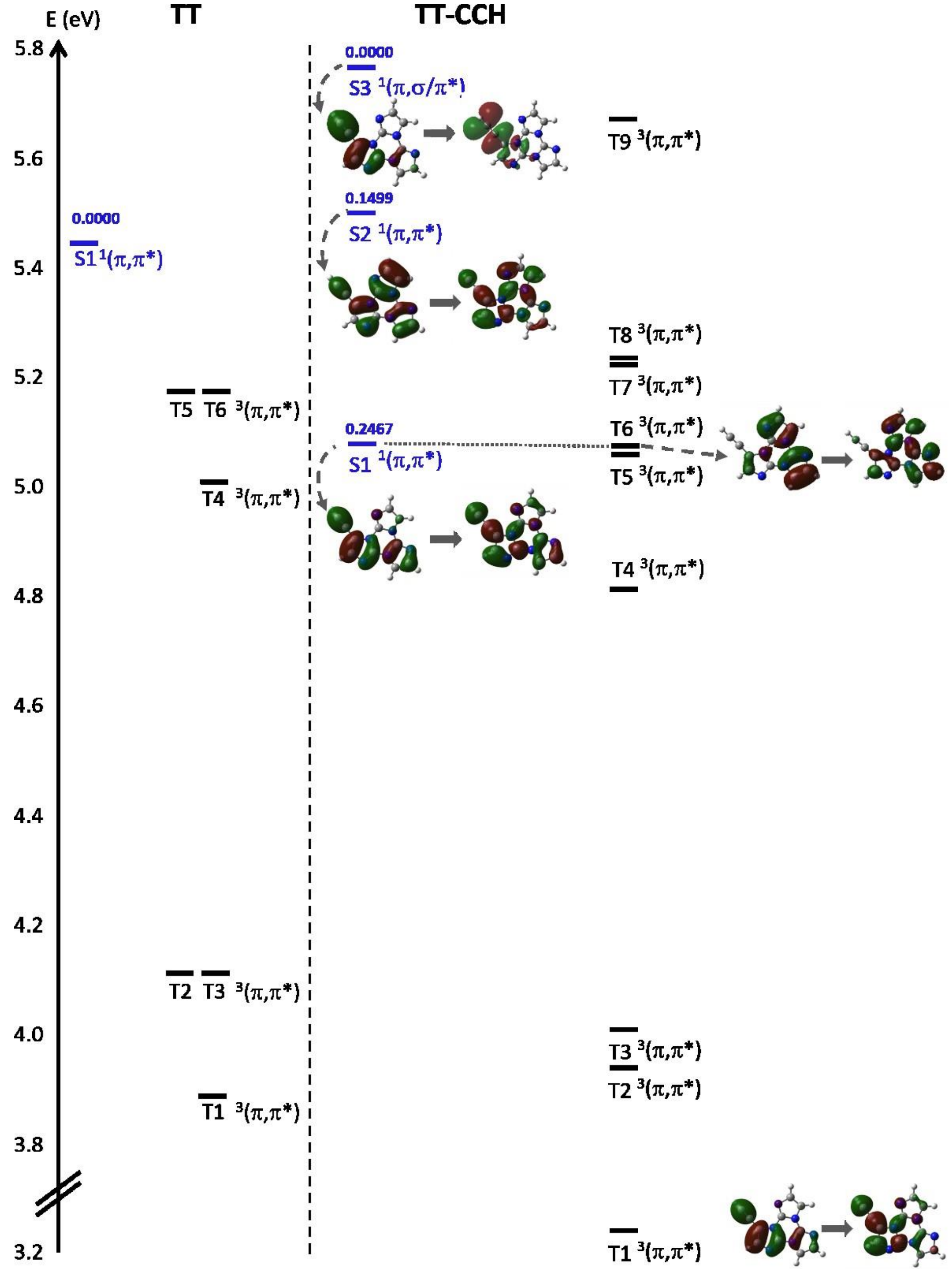

Figure S23. TD-ωB97X/6-311++G(d,p) electronic levels for **TT-CCH** at molecular level, compared with those of unsubstituted **TT**. In blue are reported the singlet levels with the corresponding values of oscillator strength *f*. Natural Transition Orbitals are included for selected transitions.

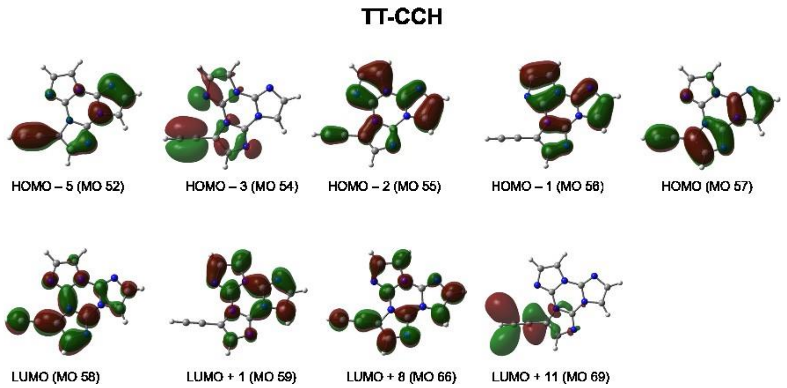

Figure S24. Plots of the ωB97X/6-311++G(d,p) MOs mainly involved in the lowest energy transitions of **TT-CCH** at the optimized ground state geometry (isosurfaces value 0.02).

Table S2 First TD-ωB97X/6-311++G(d,p) triplet and singlet electronic transitions computed for **TT-CCH** at the optimized ground state geometry.

```
Excitation energies and oscillator strengths:

 Excited State   1:      Triplet-A      3.2486 eV  381.65 nm  f=n.d.  <S**2>=2.000
      55 -> 58        0.12658
      57 -> 58        0.62532
      57 -> 66       -0.14362
      57 <- 58        0.10608

 Excited State   2:      Triplet-A      3.9434 eV  314.41 nm  f=n.d.  <S**2>=2.000
      55 -> 58        0.24487
      55 -> 66        0.17562
      56 -> 59        0.53846
      57 -> 59        0.11600
      57 -> 66       -0.14104

 Excited State   3:      Triplet-A      4.0891 eV  303.20 nm  f=n.d.  <S**2>=2.000
      50 -> 59        0.10554
      55 -> 59        0.42801
      56 -> 58        0.29856
      56 -> 66        0.26870
      57 -> 59       -0.20746

 Excited State   4:      Triplet-A      4.8239 eV  257.02 nm  f=n.d.  <S**2>=2.000
      50 -> 58        0.11664
      52 -> 58        0.18287
      52 -> 59       -0.10998
      54 -> 69       -0.16366
      55 -> 58       -0.12945
      55 -> 66        0.11151
      56 -> 58       -0.23700
      56 -> 59        0.14448
      57 -> 58        0.14648
      57 -> 59       -0.24663
      57 -> 66        0.31145
      57 -> 70       -0.13974
      57 -> 77        0.13786

 Excited State   5:      Triplet-A      5.0893 eV  243.62 nm  f=n.d.  <S**2>=2.000
      48 -> 58       -0.12214
      55 -> 58        0.10793
      55 -> 59       -0.28606
      55 -> 70       -0.15779
      56 -> 58        0.39989
      56 -> 70       -0.11775
      56 -> 72        0.14046
      57 -> 66        0.20520

 Excited State   6:      Triplet-A      5.1252 eV  241.91 nm  f=n.d.  <S**2>=2.000
      50 -> 59        0.12441
      55 -> 58       -0.31590
      55 -> 66       -0.19073
      55 -> 72       -0.11210
      56 -> 59        0.13224
      56 -> 66        0.21350
      56 -> 70        0.17978
      57 -> 59        0.31860
      57 -> 77       -0.11562

 Excited State   7:      Singlet-A      5.1257 eV  241.89 nm  f=0.2467  <S**2>=0.000
      56 -> 59       -0.19348
      57 -> 58        0.63615

 Excited State   8:      Triplet-A      5.2473 eV  236.28 nm  f=n.d.  <S**2>=2.000
      47 -> 58       -0.12308
      49 -> 69       -0.19306
```

```
      52 -> 59        -0.11295
      54 -> 69         0.41961
      54 -> 75         0.10820
      54 -> 79        -0.12099
      55 -> 58        -0.10282
      55 -> 59        -0.13182
      56 -> 59         0.11404
      57 -> 59        -0.15741
      57 -> 66         0.13619

Excited State   9:      Triplet-A      5.2837 eV  234.66 nm  f=n.d.  <S**2>=2.000
      52 -> 69        -0.21578
      54 -> 58         0.29832
      54 -> 66        -0.14310
      55 -> 69         0.16518
      57 -> 65         0.11325
      57 -> 69         0.41306
      57 -> 75         0.10163
      57 -> 79        -0.10644

Excited State  10:      Singlet-A      5.5093 eV  225.04 nm  f=0.1499  <S**2>=0.000
      55 -> 58         0.27310
      56 -> 58         0.39090
      56 -> 59         0.27620
      56 -> 66         0.12738
      57 -> 58         0.13036
      57 -> 59         0.29348
      57 -> 66        -0.14448

Excited State  11:      Triplet-A      5.6867 eV  218.02 nm  f=n.d.  <S**2>=2.000
      47 -> 58         0.10962
      47 -> 59        -0.13982
      48 -> 58        -0.11166
      52 -> 59         0.18109
      54 -> 69         0.18107
      55 -> 66         0.16159
      55 -> 70         0.12960
      56 -> 58         0.13205
      56 -> 72        -0.14933
      56 -> 77         0.15553
      57 -> 59         0.26760
      57 -> 66         0.15159
      57 -> 70        -0.19197

Excited State  12:      Singlet-A      5.7799 eV  214.51 nm  f=0.0000  <S**2>=0.000
      52 -> 69        -0.21387
      54 -> 58        -0.19256
      55 -> 69         0.18331
      57 -> 65         0.14784
      57 -> 69         0.49033
      57 -> 75         0.10982
      57 -> 79        -0.11410

Excited State  13:      Triplet-A      5.8169 eV  213.15 nm  f=n.d.  <S**2>=2.000
      52 -> 69         0.11637
      54 -> 58         0.49806
      54 -> 66        -0.18848
      55 -> 69        -0.10429
      57 -> 69        -0.28791

Excited State  14:      Singlet-A      5.8782 eV  210.92 nm  f=0.2084  <S**2>=0.000
      55 -> 58         0.22377
      55 -> 66         0.14455
      56 -> 58        -0.36812
      56 -> 59         0.47550
      57 -> 59        -0.17946

Excited State  15:      Triplet-A      5.9071 eV  209.89 nm  f=n.d.  <S**2>=2.000
      47 -> 58         0.15576
      48 -> 58         0.18283
      50 -> 58         0.11910
```

```
      50 -> 66        -0.13462
      52 -> 58         0.19788
      54 -> 69         0.18537
      55 -> 59         0.14990
      55 -> 70        -0.21757
      55 -> 72         0.11767
      56 -> 58        -0.15760
      56 -> 72         0.17110
      56 -> 77        -0.11789
      56 -> 87         0.10047
      57 -> 59         0.13401
      57 ->106         0.11428

 Excited State  16:      Singlet-A      6.0348 eV  205.45 nm  f=0.0003  <S**2>=0.000
      53 -> 58         0.11737
      54 -> 58         0.56379
      54 -> 66        -0.21685
      57 -> 69         0.17835

 Excited State  17:      Singlet-A      6.0392 eV  205.30 nm  f=0.5076  <S**2>=0.000
      55 -> 59        -0.38732
      56 -> 58        -0.29737
      57 -> 59         0.45048

 Excited State  18:      Triplet-A      6.1286 eV  202.30 nm  f=n.d.  <S**2>=2.000
      50 -> 58        -0.20930
      55 -> 58         0.33109
      55 -> 70        -0.10523
      55 -> 72        -0.11191
      55 -> 77         0.17012
      56 -> 58        -0.15760
      56 -> 59        -0.15644
      56 -> 70         0.24664
      57 -> 59         0.14654
      57 -> 66         0.13199
```

Table S3 First TD-ωB97X/6-311++G(d,p) triplet and singlet electronic transitions computed for the hydrogen bonded dimer of **TT-CCH** (Figure 6a) at the optimized ground state geometry.

```
Excitation energies and oscillator strengths:

 Excited State   1:      Triplet-A      3.2218 eV  384.83 nm  f=n.d.  <S**2>=2.000
     113 ->115        -0.43313
     113 ->129         0.11947
     114 ->116         0.44973
     114 ->127        -0.12338

 Excited State   2:      Triplet-A      3.2248 eV  384.47 nm  f=n.d.  <S**2>=2.000
     113 ->116        -0.43783
     113 ->127         0.11530
     114 ->115         0.44586
     114 ->129        -0.12603

 Excited State   3:      Triplet-A      3.9616 eV  312.97 nm  f=n.d.  <S**2>=2.000
     109 ->116         0.16929
     109 ->127         0.12687
     110 ->115        -0.16744
     110 ->129        -0.12004
     111 ->117        -0.35547
     112 ->117        -0.13365
     112 ->118         0.38450
     113 ->127        -0.10132

 Excited State   4:      Triplet-A      3.9616 eV  312.97 nm  f=n.d.  <S**2>=2.000
     109 ->115        -0.16511
     109 ->129        -0.11553
```

```
    110 ->116         0.17117
    110 ->127         0.13167
    111 ->117         0.14107
    111 ->118         0.37960
    112 ->117        -0.35663

Excited State   5:      Triplet-A      4.1119 eV  301.53 nm  f=n.d.  <S**2>=2.000
    109 ->117         0.30927
    110 ->118        -0.30736
    111 ->116        -0.20158
    111 ->127        -0.17999
    112 ->115         0.22186
    112 ->129         0.17324
    113 ->117        -0.13228
    114 ->118         0.12405

Excited State   6:      Triplet-A      4.1120 eV  301.52 nm  f=n.d.  <S**2>=2.000
    109 ->118        -0.29929
    110 ->117         0.31733
    111 ->115         0.22236
    111 ->129         0.17263
    112 ->116        -0.20273
    112 ->127        -0.18121
    113 ->118         0.14450
    114 ->117        -0.11187

Excited State   7:      Triplet-A      4.8011 eV  258.24 nm  f=n.d.  <S**2>=2.000
    105 ->115         0.12447
    105 ->118         0.10115
    106 ->116        -0.15836
    108 ->135        -0.14251
    111 ->115        -0.11862
    111 ->118        -0.11192
    112 ->116         0.13526
    113 ->118         0.21427
    113 ->129         0.19310
    114 ->116        -0.11655
    114 ->117        -0.15175
    114 ->127        -0.26244
    114 ->139         0.11498

Excited State   8:      Triplet-A      4.8114 eV  257.69 nm  f=n.d.  <S**2>=2.000
    105 ->116        -0.14756
    106 ->115         0.13178
    106 ->118         0.10842
    111 ->116         0.14246
    112 ->115        -0.11777
    112 ->118        -0.10977
    113 ->116        -0.12721
    113 ->117        -0.15549
    113 ->127        -0.25672
    113 ->139         0.11203
    114 ->118         0.22983
    114 ->129         0.20745
    114 ->147         0.10093
    114 ->150        -0.10132

Excited State   9:      Singlet-A      5.0702 eV  244.54 nm  f=0.7426  <S**2>=0.000
    112 ->117         0.10438
    113 ->115        -0.44113
    113 ->118        -0.11037
    114 ->116         0.47680

Excited State  10:      Triplet-A      5.0710 eV  244.49 nm  f=n.d.  <S**2>=2.000
    109 ->118         0.19797
    110 ->117        -0.18563
    110 ->139         0.13657
    111 ->115         0.30164
    111 ->142        -0.10577
    112 ->116        -0.27009
    112 ->139         0.13492
```

```
    113 ->129         0.11202
    114 ->127        -0.11753

Excited State  11:      Triplet-A      5.0716 eV  244.47 nm  f=n.d.  <S**2>=2.000
    109 ->116        -0.10445
    109 ->117        -0.18188
    109 ->139         0.13761
    110 ->118         0.20231
    110 ->129        -0.10205
    111 ->116        -0.26875
    111 ->139         0.13890
    112 ->115         0.30081
    112 ->142        -0.10593
    113 ->127        -0.12868
    114 ->129         0.11104

Excited State  12:      Singlet-A      5.1017 eV  243.03 nm  f=0.0000  <S**2>=0.000
    111 ->117         0.14068
    112 ->118        -0.13484
    113 ->116         0.44890
    114 ->115        -0.44126
    114 ->118        -0.12170

Excited State  13:      Triplet-A      5.1109 eV  242.59 nm  f=n.d.  <S**2>=2.000
    108 ->135        -0.13801
    109 ->115        -0.22307
    110 ->116         0.21059
    111 ->129         0.15491
    112 ->127        -0.13567
    112 ->139        -0.12489
    113 ->118        -0.22341
    114 ->117         0.22355

Excited State  14:      Triplet-A      5.1175 eV  242.27 nm  f=n.d.  <S**2>=2.000
    109 ->116         0.21500
    109 ->127         0.11236
    110 ->115        -0.25036
    111 ->117         0.10823
    111 ->127        -0.14091
    111 ->139        -0.13078
    112 ->118        -0.10478
    112 ->129         0.16296
    113 ->117         0.22444
    114 ->118        -0.20220

Excited State  15:      Triplet-A      5.2252 eV  237.28 nm  f=n.d.  <S**2>=2.000
    107 ->138         0.13270
    107 ->144        -0.18881
    108 ->131        -0.10717
    108 ->135        -0.34396
    108 ->136         0.12979
    109 ->115         0.13518
    110 ->117         0.11116

Excited State  16:      Triplet-A      5.2683 eV  235.34 nm  f=n.d.  <S**2>=2.000
    105 ->144        -0.10193
    106 ->135        -0.15879
    107 ->115         0.20870
    107 ->129        -0.10382
    108 ->116        -0.26029
    108 ->127         0.14173
    110 ->135         0.10181
    113 ->138        -0.13751
    113 ->144         0.18481
    114 ->131         0.10234
    114 ->135         0.32266
    114 ->136        -0.11529

Excited State  17:      Triplet-A      5.2962 eV  234.10 nm  f=n.d.  <S**2>=2.000
     97 ->135        -0.12698
    107 ->135         0.29470
```

```
     107 ->136        -0.11365
     108 ->138        -0.16523
     108 ->144         0.23116
     109 ->117        -0.10314
     113 ->117        -0.12255
     114 ->118         0.12901

 Excited State  18:      Triplet-A      5.3048 eV  233.72 nm  f=n.d.  <S**2>=2.000
     105 ->135        -0.15345
     106 ->144        -0.10306
     107 ->116        -0.22082
     107 ->127         0.11807
     108 ->115         0.26278
     108 ->129        -0.12793
     109 ->135         0.10482
     113 ->135         0.29771
     113 ->136        -0.10761
     114 ->138        -0.14927
     114 ->144         0.19895

 Excited State  19:      Singlet-A      5.4935 eV  225.69 nm  f=0.3116  <S**2>=0.000
     109 ->115         0.17072
     110 ->116        -0.18558
     111 ->115         0.28406
     111 ->118        -0.18494
     112 ->116        -0.20783
     112 ->117         0.26150
     113 ->115         0.10250
     113 ->118        -0.22809
     114 ->117         0.21563
     114 ->127         0.12594

 Excited State  20:      Singlet-A      5.5081 eV  225.09 nm  f=0.0000  <S**2>=0.000
     109 ->116        -0.18648
     110 ->115         0.18875
     111 ->116        -0.23282
     111 ->117         0.22717
     112 ->115         0.29800
     112 ->118        -0.15557
     113 ->117         0.21401
     113 ->127         0.11995
     114 ->115         0.13541
     114 ->118        -0.20969

 Excited State  21:      Triplet-A      5.6408 eV  219.80 nm  f=n.d.  <S**2>=2.000
     105 ->118         0.12826
     106 ->117        -0.14304
     108 ->135        -0.17718
     109 ->129        -0.11237
     110 ->127         0.10873
     113 ->118         0.18764
     113 ->137         0.12369
     114 ->117        -0.23637
     114 ->127         0.13078
     114 ->139        -0.14240

 Excited State  22:      Triplet-A      5.6641 eV  218.90 nm  f=n.d.  <S**2>=2.000
     105 ->117        -0.12950
     106 ->118         0.12893
     107 ->135        -0.16687
     108 ->144        -0.12890
     109 ->127         0.10631
     110 ->129        -0.10565
     111 ->140        -0.10220
     111 ->151        -0.10010
     112 ->142        -0.10835
     113 ->117        -0.21779
     113 ->127         0.12198
     113 ->139        -0.14154
     114 ->118         0.17587
     114 ->129        -0.10750
```

```
     114 ->137         0.11991

 Excited State  23:      Triplet-A      5.7778 eV  214.59 nm  f=n.d.  <S**2>=2.000
     107 ->115        -0.29946
     107 ->129         0.12500
     108 ->116         0.37077
     108 ->127        -0.17271
     113 ->138        -0.11359
     113 ->144         0.13947
     114 ->135         0.25671

 Excited State  24:      Singlet-A      5.7782 eV  214.57 nm  f=0.0000  <S**2>=0.000
     106 ->135        -0.15028
     107 ->115        -0.17439
     108 ->116         0.21935
     108 ->127        -0.10788
     110 ->135         0.10621
     113 ->138        -0.16352
     113 ->144         0.20058
     114 ->131         0.11968
     114 ->135         0.37042
     114 ->136        -0.12939

 Excited State  25:      Triplet-A      5.7982 eV  213.83 nm  f=n.d.  <S**2>=2.000
     107 ->116        -0.30242
     107 ->127         0.13541
     108 ->115         0.36502
     108 ->129        -0.15041
     113 ->135        -0.24658
     114 ->138         0.12661
     114 ->144        -0.15351
```

Table S4 First TD-ωB97X/6-311++G(d,p) triplet and singlet electronic transitions computed for the hydrogen bonded tetramer of **TT-CCH** (Figure 6b) at the optimized ground state geometry.

```
Excitation energies and oscillator strengths:

 Excited State   1:      Triplet-A      3.2221 eV  384.79 nm  f=n.d.  <S**2>=2.000
     225 -> 231       -0.11473
     225 -> 232       -0.11715
     226 -> 231        0.14606
     226 -> 232        0.11314
     227 -> 230       -0.11359
     227 -> 231        0.24693
     227 -> 232        0.27163
     228 -> 231        0.29221
     228 -> 232        0.24174

 Excited State   2:      Triplet-A      3.2221 eV  384.79 nm  f=n.d.  <S**2>=2.000
     225 -> 231       -0.13252
     225 -> 232        0.10144
     226 -> 231       -0.12662
     226 -> 232        0.13026
     227 -> 230        0.10245
     227 -> 231        0.29894
     227 -> 232       -0.24644
     228 -> 230       -0.10889
     228 -> 231       -0.24135
     228 -> 232        0.26527

 Excited State   3:      Triplet-A      3.2375 eV  382.96 nm  f=n.d.  <S**2>=2.000
     225 -> 230        0.33500
     225 -> 232        0.19204
     226 -> 229        0.34829
     226 -> 231       -0.11406
     226 -> 249       -0.10107
     227 -> 230        0.19767
```

```
    228 -> 229       -0.21761

Excited State   4:      Triplet-A      3.2377 eV  382.94 nm  f=n.d.  <S**2>=2.000
    225 -> 229        0.36273
    225 -> 231       -0.11526
    225 -> 249       -0.10666
    226 -> 230        0.32057
    226 -> 232        0.18681
    227 -> 229        0.20219
    228 -> 230       -0.21139

Excited State   5:      Triplet-A      3.9432 eV  314.42 nm  f=n.d.  <S**2>=2.000
    219 -> 229       -0.14342
    220 -> 230       -0.12444
    223 -> 238        0.36631
    224 -> 233       -0.12932
    224 -> 235       -0.33946
    226 -> 235        0.10931

Excited State   6:      Triplet-A      3.9444 eV  314.33 nm  f=n.d.  <S**2>=2.000
    219 -> 230        0.11588
    220 -> 229        0.15625
    220 -> 249        0.10038
    223 -> 233       -0.13037
    223 -> 235       -0.34268
    224 -> 238        0.36549
    226 -> 238       -0.10543

Excited State   7:      Triplet-A      3.9617 eV  312.95 nm  f=n.d.  <S**2>=2.000
    217 -> 231       -0.10116
    218 -> 231        0.11136
    218 -> 232       -0.10284
    222 -> 233        0.34583
    222 -> 234        0.36550
    222 -> 235       -0.12057

Excited State   8:      Triplet-A      3.9617 eV  312.95 nm  f=n.d.  <S**2>=2.000
    217 -> 231       -0.10278
    218 -> 231       -0.10941
    218 -> 232       -0.10458
    221 -> 233       -0.34852
    221 -> 234        0.36287
    221 -> 235        0.12052

Excited State   9:      Triplet-A      4.1112 eV  301.58 nm  f=n.d.  <S**2>=2.000
    217 -> 235        0.14092
    219 -> 235       -0.25884
    220 -> 230       -0.11706
    220 -> 238       -0.27557
    223 -> 230        0.19146
    223 -> 257        0.15024
    224 -> 229       -0.20211
    224 -> 235       -0.10171
    224 -> 249       -0.16486
    225 -> 238       -0.10700

Excited State  10:      Triplet-A      4.1123 eV  301.49 nm  f=n.d.  <S**2>=2.000
    217 -> 238       -0.11895
    218 -> 235        0.11274
    219 -> 230        0.10894
    219 -> 238        0.25194
    220 -> 233        0.11114
    220 -> 235        0.26338
    223 -> 229       -0.19427
    223 -> 249       -0.15811
    224 -> 230        0.18705
    224 -> 257        0.14511
    225 -> 235        0.10314

Excited State  11:      Triplet-A      4.1125 eV  301.48 nm  f=n.d.  <S**2>=2.000
    217 -> 233       -0.24987
```

```
     218 -> 234        0.28328
     219 -> 233       -0.13643
     220 -> 234       -0.10119
     221 -> 232       -0.14628
     221 -> 253       -0.12433
     222 -> 231        0.19431
     222 -> 232       -0.12073
     222 -> 253       -0.10277
     222 -> 256       -0.15416
     227 -> 234       -0.10640

 Excited State  12:      Triplet-A      4.1125 eV  301.48 nm  f=n.d.  <S**2>=2.000
     217 -> 234       -0.24460
     218 -> 233        0.26717
     219 -> 234       -0.15699
     220 -> 235        0.10748
     221 -> 231        0.18847
     221 -> 232        0.11891
     221 -> 253        0.10103
     221 -> 256       -0.15017
     222 -> 232       -0.13963
     222 -> 253       -0.11880
     228 -> 234        0.10084

 Excited State  13:      Triplet-A      4.7784 eV  259.47 nm  f=n.d.  <S**2>=2.000
     211 -> 229        0.10386
     223 -> 229        0.12547
     224 -> 238        0.12420
     225 -> 229       -0.11613
     225 -> 235        0.14780
     225 -> 249       -0.18668
     226 -> 238        0.14382
     226 -> 253       -0.10057
     226 -> 257       -0.18894
     227 -> 249       -0.12030
     228 -> 253        0.10066

 Excited State  14:      Triplet-A      4.7788 eV  259.45 nm  f=n.d.  <S**2>=2.000
     210 -> 229       -0.10331
     223 -> 238        0.11732
     224 -> 229        0.13082
     225 -> 238       -0.15278
     225 -> 253        0.10648
     225 -> 257        0.19487
     226 -> 229        0.10713
     226 -> 235       -0.13812
     226 -> 249        0.17952
     228 -> 249       -0.12878

 Excited State  15:      Triplet-A      4.8076 eV  257.89 nm  f=n.d.  <S**2>=2.000
     221 -> 231        0.12539
     226 -> 253       -0.11529
     227 -> 233        0.16313
     227 -> 256        0.16954
     228 -> 234       -0.14676
     228 -> 253       -0.16793

 Excited State  16:      Triplet-A      4.8076 eV  257.89 nm  f=n.d.  <S**2>=2.000
     209 -> 231        0.10085
     222 -> 231        0.12537
     225 -> 253       -0.10778
     227 -> 234        0.15075
     227 -> 253        0.17290
     228 -> 233       -0.15954
     228 -> 256       -0.16599

 Excited State  17:      Triplet-A      5.0381 eV  246.09 nm  f=n.d.  <S**2>=2.000
     219 -> 235        0.15170
     220 -> 238        0.19039
     223 -> 230        0.25432
     223 -> 232        0.10506
```

```
     223 -> 295       -0.10143
     224 -> 229       -0.26096
     224 -> 235       -0.11258
     225 -> 238        0.10847
     226 -> 235        0.10931
     226 -> 249        0.11192

 Excited State  18:      Triplet-A      5.0386 eV  246.07 nm  f=n.d.  <S**2>=2.000
     219 -> 238        0.17416
     220 -> 235        0.16370
     223 -> 229        0.25708
     223 -> 235        0.11638
     224 -> 230       -0.25798
     224 -> 232       -0.10784
     224 -> 295        0.10027
     225 -> 235        0.10320
     225 -> 249        0.10894
     226 -> 238        0.10960

 Excited State  19:      Singlet-A      5.0592 eV  245.07 nm  f=1.6383  <S**2>=0.000
     225 -> 230        0.31652
     226 -> 229        0.31614
     227 -> 232        0.32738
     228 -> 231        0.31337

 Excited State  20:      Triplet-A      5.0698 eV  244.55 nm  f=n.d.  <S**2>=2.000
     217 -> 234       -0.13409
     218 -> 233        0.14091
     218 -> 234        0.10478
     221 -> 231       -0.11623
     222 -> 230       -0.13818
     222 -> 231       -0.25193
     222 -> 232        0.25426
     222 -> 273       -0.10049

 Excited State  21:      Triplet-A      5.0698 eV  244.55 nm  f=n.d.  <S**2>=2.000
     217 -> 233       -0.13070
     218 -> 233       -0.10216
     218 -> 234        0.14441
     221 -> 230       -0.13915
     221 -> 231        0.25166
     221 -> 232        0.25451
     221 -> 273       -0.10050
     222 -> 231       -0.11631

 Excited State  22:      Singlet-A      5.0765 eV  244.23 nm  f=0.0000  <S**2>=0.000
     225 -> 229       -0.29703
     226 -> 230       -0.28977
     227 -> 231        0.33742
     228 -> 232        0.34236

 Excited State  23:      Singlet-A      5.1004 eV  243.09 nm  f=0.0029  <S**2>=0.000
     225 -> 230        0.16797
     225 -> 232        0.24857
     226 -> 229        0.21578
     226 -> 231       -0.22444
     227 -> 230        0.24280
     227 -> 232       -0.20562
     228 -> 229       -0.21593
     228 -> 231       -0.23642

 Excited State  24:      Singlet-A      5.1012 eV  243.05 nm  f=0.0000  <S**2>=0.000
     223 -> 235       -0.10391
     225 -> 229        0.25646
     225 -> 231       -0.20736
     226 -> 230        0.18085
     226 -> 232        0.25561
     227 -> 229        0.21289
     227 -> 231        0.21604
     228 -> 230       -0.25077
     228 -> 232        0.16223
```

```
Excited State  25:      Triplet-A      5.1107 eV  242.60 nm  f=n.d.  <S**2>=2.000
    217 -> 230       -0.13155
    218 -> 231        0.10348
    219 -> 230        0.11808
    219 -> 232        0.12904
    220 -> 229        0.17334
    223 -> 249        0.10808
    225 -> 233        0.10594
    225 -> 235        0.10648
    226 -> 238        0.13498
    227 -> 235        0.10313
    228 -> 234        0.12758

Excited State  26:      Triplet-A      5.1114 eV  242.56 nm  f=n.d.  <S**2>=2.000
    217 -> 229        0.11969
    217 -> 231        0.10709
    218 -> 230       -0.11390
    218 -> 232        0.10586
    219 -> 229       -0.12687
    219 -> 231        0.11024
    220 -> 230       -0.12153
    220 -> 232       -0.11529
    225 -> 238       -0.12184
    226 -> 233       -0.11655
    227 -> 234        0.14643
    228 -> 233       -0.10318
    228 -> 235        0.10893

Excited State  27:      Triplet-A      5.1159 eV  242.35 nm  f=n.d.  <S**2>=2.000
    217 -> 232       -0.16950
    218 -> 231       -0.17558
    219 -> 230        0.16774
    220 -> 229        0.17167
    225 -> 235        0.11914
    226 -> 234       -0.10293
    227 -> 233        0.15798
    228 -> 234       -0.13820

Excited State  28:      Triplet-A      5.1164 eV  242.33 nm  f=n.d.  <S**2>=2.000
    217 -> 231       -0.15645
    218 -> 232       -0.15907
    219 -> 229       -0.18387
    220 -> 230       -0.18275
    226 -> 235       -0.13399
    227 -> 234       -0.12567
    228 -> 233        0.14451

Excited State  29:      Triplet-A      5.2195 eV  237.54 nm  f=n.d.  <S**2>=2.000
    213 -> 287       -0.10609
    214 -> 286       -0.10103
    215 -> 264        0.12814
    215 -> 265       -0.21539
    216 -> 264        0.20961

Excited State  30:      Triplet-A      5.2195 eV  237.54 nm  f=n.d.  <S**2>=2.000
    214 -> 287        0.10896
    215 -> 264        0.20962
    216 -> 264       -0.12825
    216 -> 265       -0.21540

Excited State  31:      Triplet-A      5.2691 eV  235.31 nm  f=n.d.  <S**2>=2.000
    214 -> 230       -0.10197
    215 -> 231       -0.12590
    215 -> 232        0.13493
    216 -> 231       -0.10496
    216 -> 232        0.11939
    227 -> 264       -0.15716
    227 -> 265        0.14403
    228 -> 264        0.14710
    228 -> 265       -0.14301
```

```
Excited State  32:      Triplet-A      5.2691 eV  235.30 nm  f=n.d.  <S**2>=2.000
    213 -> 230        -0.10342
    215 -> 231        -0.10514
    215 -> 232        -0.11973
    216 -> 231         0.12561
    216 -> 232         0.13502
    227 -> 264        -0.14558
    227 -> 265        -0.14127
    228 -> 264        -0.15882
    228 -> 265        -0.14556

Excited State  33:      Triplet-A      5.2945 eV  234.18 nm  f=n.d.  <S**2>=2.000
    213 -> 264        -0.14665
    213 -> 265         0.13636
    214 -> 264         0.14830
    214 -> 265        -0.14394
    215 -> 274         0.10334
    215 -> 279         0.10458
    215 -> 286        -0.10630
    215 -> 287         0.10728

Excited State  34:      Triplet-A      5.2946 eV  234.17 nm  f=n.d.  <S**2>=2.000
    213 -> 264         0.14849
    213 -> 265         0.14408
    214 -> 264         0.14654
    214 -> 265         0.13609
    216 -> 274        -0.10302
    216 -> 279         0.10455
    216 -> 287        -0.11370

Excited State  35:      Triplet-A      5.3059 eV  233.67 nm  f=n.d.  <S**2>=2.000
    213 -> 232         0.10006
    214 -> 232        -0.10495
    215 -> 229         0.12294
    215 -> 230         0.13301
    216 -> 229         0.10466
    216 -> 230         0.11616
    225 -> 264        -0.14125
    225 -> 265         0.13323
    226 -> 264        -0.13999
    226 -> 265         0.13536

Excited State  36:      Triplet-A      5.3060 eV  233.67 nm  f=n.d.  <S**2>=2.000
    213 -> 232         0.10497
    214 -> 232         0.10024
    215 -> 229        -0.10290
    215 -> 230         0.11735
    216 -> 229         0.12153
    216 -> 230        -0.13490
    225 -> 264         0.13942
    225 -> 265         0.13486
    226 -> 264        -0.14196
    226 -> 265        -0.13364

Excited State  37:      Singlet-A      5.4820 eV  226.17 nm  f=0.0000  <S**2>=0.000
    219 -> 230         0.13991
    220 -> 229         0.18559
    223 -> 229        -0.20941
    223 -> 235        -0.21644
    224 -> 230         0.24441
    224 -> 238         0.16706
    225 -> 235        -0.16309
    226 -> 230        -0.12213
    226 -> 238        -0.16542
    227 -> 235        -0.10388

Excited State  38:      Singlet-A      5.4838 eV  226.09 nm  f=0.6481  <S**2>=0.000
    219 -> 229        -0.13673
    220 -> 230        -0.12572
    221 -> 234        -0.11674
```

```
     222 -> 231       -0.10692
     222 -> 233       -0.10446
     223 -> 230        0.19414
     223 -> 238        0.16115
     224 -> 229       -0.16464
     224 -> 235       -0.18989
     225 -> 238        0.16460
     226 -> 233        0.12740
     226 -> 235        0.16327
     227 -> 234       -0.11853
     228 -> 235       -0.13043

 Excited State  39:      Singlet-A      5.5039 eV  225.27 nm  f=0.0000  <S**2>=0.000
     217 -> 232        0.15269
     218 -> 231        0.16546
     221 -> 231        0.20412
     221 -> 232        0.10422
     221 -> 233        0.14968
     222 -> 230        0.11792
     222 -> 231        0.11548
     222 -> 232       -0.18412
     222 -> 234        0.17247
     226 -> 234       -0.11118
     227 -> 233        0.18074
     228 -> 234       -0.16834

 Excited State  40:      Singlet-A      5.5060 eV  225.18 nm  f=0.0269  <S**2>=0.000
     217 -> 231       -0.14475
     218 -> 232       -0.14898
     219 -> 229       -0.12191
     220 -> 230       -0.12295
     221 -> 230        0.10215
     221 -> 231       -0.10323
     221 -> 232       -0.16627
     221 -> 234        0.14869
     222 -> 231        0.18288
     222 -> 233        0.12835
     223 -> 230        0.14490
     224 -> 229       -0.13275
     226 -> 235        0.13882
     227 -> 234        0.14859
     228 -> 233       -0.16754

 Excited State  41:      Triplet-A      5.6432 eV  219.71 nm  f=n.d.  <S**2>=2.000
     215 -> 264       -0.11893
     216 -> 265        0.11244
     226 -> 238        0.10800
     227 -> 233       -0.12366
     227 -> 235        0.11695
     228 -> 234        0.16474

 Excited State  42:      Triplet-A      5.6435 eV  219.69 nm  f=n.d.  <S**2>=2.000
     215 -> 265        0.11231
     216 -> 264       -0.11871
     225 -> 238        0.11154
     227 -> 234       -0.16874
     228 -> 233        0.12176
     228 -> 235       -0.11920
```

Table S5 First TD-ωB97X/6-311++G(d,p) triplet and singlet electronic transitions computed for the π···π dimer of **TT-CCH** (Figure 6c) at the optimized ground state geometry.

```
Excitation energies and oscillator strengths:

 Excited State   1:      Triplet-A      3.2352 eV  383.23 nm  f=n.d.  <S**2>=2.000
     109 ->115        -0.10924
     113 ->115         0.42638
     113 ->116         0.22274
     114 ->115        -0.37909
     114 ->116        -0.13068
```

```
Excited State   2:      Triplet-A      3.2508 eV  381.40 nm  f=n.d.  <S**2>=2.000
    113 ->115       -0.18184
    113 ->116        0.30640
    114 ->115       -0.18664
    114 ->116        0.47296

Excited State   3:      Triplet-A      3.9357 eV  315.02 nm  f=n.d.  <S**2>=2.000
    109 ->116        0.16651
    110 ->115       -0.12474
    111 ->120        0.28394
    112 ->117        0.37028
    112 ->120        0.25213

Excited State   4:      Triplet-A      3.9464 eV  314.17 nm  f=n.d.  <S**2>=2.000
    109 ->115       -0.16471
    109 ->120        0.10403
    110 ->115        0.12650
    110 ->116        0.11767
    111 ->117        0.39249
    111 ->120       -0.19552
    112 ->120        0.27089

Excited State   5:      Triplet-A      4.0809 eV  303.82 nm  f=n.d.  <S**2>=2.000
    109 ->117        0.27680
    110 ->120        0.26966
    111 ->115       -0.18976
    111 ->131       -0.11700
    112 ->116        0.20927
    112 ->126       -0.13961
    113 ->117        0.17296
    114 ->120        0.13936

Excited State   6:      Triplet-A      4.0956 eV  302.72 nm  f=n.d.  <S**2>=2.000
    109 ->120        0.25003
    110 ->117        0.31376
    111 ->115        0.14673
    111 ->116        0.16056
    111 ->126       -0.14000
    112 ->115       -0.18196
    112 ->131       -0.12555
    113 ->120        0.16309
    114 ->117        0.12848

Excited State   7:      Triplet-A      4.7964 eV  258.49 nm  f=n.d.  <S**2>=2.000
    103 ->115       -0.11661
    107 ->135        0.12350
    110 ->115       -0.12753
    111 ->115        0.20713
    113 ->115        0.12242
    113 ->117        0.16961
    113 ->120       -0.12037
    113 ->126       -0.12639
    113 ->131        0.12688
    113 ->137       -0.12279
    114 ->117       -0.12118
    114 ->120        0.10267
    114 ->131       -0.11256

Excited State   8:      Triplet-A      4.7995 eV  258.33 nm  f=n.d.  <S**2>=2.000
    100 ->116       -0.10304
    104 ->116        0.10636
    111 ->116        0.11131
    112 ->115       -0.10785
    112 ->116        0.21965
    113 ->120       -0.11478
    114 ->117       -0.15142
    114 ->120       -0.14106
    114 ->126        0.14583
    114 ->138       -0.12481
```

```
Excited State   9:      Triplet-A      5.0565 eV  245.20 nm  f=n.d.  <S**2>=2.000
    109 ->117        -0.16025
    110 ->115         0.16658
    110 ->117         0.13275
    110 ->120        -0.12309
    110 ->137        -0.12774
    111 ->115        -0.30096
    111 ->137         0.12444
    112 ->115         0.15315
    112 ->116         0.14072

Excited State  10:      Singlet-A      5.0597 eV  245.04 nm  f=0.0129  <S**2>=0.000
    111 ->117        -0.11503
    113 ->115         0.46061
    114 ->115        -0.22434
    114 ->116        -0.39484

Excited State  11:      Triplet-A      5.0636 eV  244.85 nm  f=n.d.  <S**2>=2.000
    109 ->116        -0.12458
    109 ->120        -0.13760
    110 ->116        -0.12694
    110 ->117        -0.12250
    111 ->116         0.14161
    112 ->115        -0.19771
    112 ->116         0.30054
    112 ->138         0.17085
    112 ->147        -0.10150
    114 ->126        -0.11356

Excited State  12:      Triplet-A      5.0949 eV  243.35 nm  f=n.d.  <S**2>=2.000
    109 ->116        -0.14142
    109 ->120         0.11670
    109 ->131         0.10326
    110 ->116        -0.12037
    113 ->117         0.11551
    113 ->120         0.15162
    114 ->117         0.16640
    114 ->120         0.16042

Excited State  13:      Triplet-A      5.1180 eV  242.25 nm  f=n.d.  <S**2>=2.000
    109 ->115        -0.15287
    110 ->115         0.20248
    111 ->117        -0.10943
    111 ->131         0.12225
    113 ->117         0.19242
    113 ->120        -0.13378
    114 ->117        -0.16162
    114 ->120         0.14230

Excited State  14:      Singlet-A      5.1229 eV  242.02 nm  f=0.3578  <S**2>=0.000
    111 ->120        -0.12377
    112 ->117        -0.14888
    113 ->116        -0.37015
    114 ->115         0.39416
    114 ->116        -0.31904

Excited State  15:      Triplet-A      5.2350 eV  236.84 nm  f=n.d.  <S**2>=2.000
     97 ->135        -0.13096
    107 ->132        -0.12655
    107 ->134         0.16145
    107 ->135        -0.28527
    108 ->135        -0.11313
    108 ->136         0.11412
    114 ->117        -0.10279

Excited State  16:      Triplet-A      5.2408 eV  236.58 nm  f=n.d.  <S**2>=2.000
     98 ->134        -0.10326
    107 ->135        -0.17946
    108 ->133        -0.15060
    108 ->134         0.23617
    108 ->136        -0.20233
```

```
     109 ->117        -0.10010
     113 ->117         0.10449
     114 ->120         0.11836

 Excited State  17:      Triplet-A      5.2783 eV  234.89 nm  f=n.d.  <S**2>=2.000
     103 ->135        -0.13348
     104 ->135         0.10136
     107 ->115         0.26997
     107 ->116         0.13308
     109 ->135         0.10486
     113 ->132        -0.12373
     113 ->134         0.10795
     113 ->135        -0.25469
     114 ->134        -0.12359
     114 ->135         0.20076

 Excited State  18:      Triplet-A      5.2899 eV  234.38 nm  f=n.d.  <S**2>=2.000
     108 ->115        -0.10675
     108 ->116         0.25873
     113 ->133        -0.10436
     113 ->134         0.15661
     113 ->136        -0.13298
     114 ->133        -0.13990
     114 ->134         0.19394
     114 ->135         0.13468
     114 ->136        -0.19507

 Excited State  19:      Singlet-A      5.4563 eV  227.23 nm  f=0.0038  <S**2>=0.000
     109 ->115        -0.11835
     110 ->115         0.11788
     110 ->116         0.17888
     111 ->115        -0.16762
     111 ->116        -0.16956
     111 ->117         0.21407
     112 ->115         0.27540
     112 ->116        -0.18828
     112 ->120         0.20490
     113 ->120         0.14415
     114 ->117         0.20901

 Excited State  20:      Singlet-A      5.5007 eV  225.40 nm  f=0.2488  <S**2>=0.000
     109 ->116        -0.14552
     110 ->115         0.20987
     111 ->115        -0.27909
     111 ->120        -0.16095
     112 ->116         0.29867
     112 ->117        -0.18989
     113 ->117        -0.19549
     114 ->115        -0.10450
     114 ->120        -0.20074

 Excited State  21:      Triplet-A      5.6720 eV  218.59 nm  f=n.d.  <S**2>=2.000
     103 ->120        -0.10357
     104 ->117        -0.11348
     107 ->135        -0.12238
     112 ->115         0.10564
     113 ->117        -0.10118
     113 ->120         0.16991
     113 ->137        -0.13367
     114 ->117         0.16370
     114 ->137         0.10218

 Excited State  22:      Triplet-A      5.6778 eV  218.37 nm  f=n.d.  <S**2>=2.000
     104 ->120        -0.10287
     112 ->116        -0.10150
     112 ->147        -0.12063
     113 ->117         0.15650
     113 ->138        -0.13045
     114 ->120         0.16829
     114 ->138        -0.13356
```

```
 Excited State  23:      Singlet-A      5.7678 eV  214.96 nm  f=0.0003  <S**2>=0.000
     104 ->135         0.11568
     107 ->115        -0.16874
     110 ->135        -0.10207
     113 ->129         0.10046
     113 ->134         0.20510
     113 ->135        -0.21661
     114 ->132         0.15620
     114 ->135         0.28366
     114 ->136        -0.12094

 Excited State  24:      Singlet-A      5.7759 eV  214.66 nm  f=0.0010  <S**2>=0.000
     103 ->135         0.10276
     104 ->134         0.10356
     108 ->116        -0.13206
     113 ->132         0.12897
     113 ->135         0.21915
     113 ->136        -0.15486
     114 ->133        -0.16552
     114 ->134         0.28471
     114 ->136        -0.19563

 Excited State  25:      Triplet-A      5.7949 eV  213.96 nm  f=n.d.  <S**2>=2.000
     107 ->115         0.41800
     107 ->116         0.18671
     107 ->131        -0.10661
     113 ->135         0.17152
     114 ->135        -0.15338

 Excited State  26:      Singlet-A      5.8065 eV  213.53 nm  f=0.0035  <S**2>=0.000
     109 ->115        -0.14791
     109 ->120         0.14310
     111 ->115         0.16293
     111 ->116         0.27486
     111 ->117         0.36074
     112 ->115        -0.25629
     112 ->116         0.12322
     112 ->120         0.26030

 Excited State  27:      Triplet-A      5.8274 eV  212.76 nm  f=n.d.  <S**2>=2.000
     108 ->115        -0.20450
     108 ->116         0.43991
     108 ->129        -0.10169
     114 ->134        -0.15024
     114 ->136         0.13301

 Excited State  28:      Singlet-A      5.8466 eV  212.06 nm  f=0.3741  <S**2>=0.000
     109 ->116         0.15800
     110 ->115        -0.13510
     111 ->115        -0.24195
     111 ->120         0.27704
     112 ->116         0.20244
     112 ->117         0.33195
     112 ->120         0.14362
     113 ->117        -0.16135
     114 ->120        -0.13795

 Excited State  29:      Triplet-A      5.8915 eV  210.45 nm  f=n.d.  <S**2>=2.000
      96 ->115        -0.13383
     103 ->115        -0.10475
     104 ->115         0.11586
     107 ->135        -0.15344
     110 ->120         0.10740
     110 ->137        -0.14781
     111 ->115         0.12645

 Excited State  30:      Triplet-A      5.8948 eV  210.33 nm  f=n.d.  <S**2>=2.000
      94 ->116        -0.11753
      96 ->116         0.13502
     100 ->116         0.10949
     104 ->116        -0.12586
```

```
    108 ->134         0.10998
    108 ->136        -0.10408
    109 ->138        -0.13599
    110 ->138        -0.12901
    112 ->138         0.11751
    112 ->147        -0.11652
    114 ->117        -0.10923
```

Table S6 First TD-ωB97X/6-311++G(d,p) triplet and singlet electronic transitions computed for the π···π tetramer of **TT-CCH** (Figure 6d) at the optimized ground state geometry.

```
Excitation energies and oscillator strengths:

 Excited State   1:      Triplet-A      3.2367 eV  383.06 nm  f=n.d.  <S**2>=2.000
     225 -> 229       0.17936
     225 -> 230       0.25012
     225 -> 231      -0.22809
     225 -> 232       0.10529
     226 -> 229      -0.17928
     226 -> 230      -0.20565
     227 -> 229       0.27545
     227 -> 230       0.15009
     228 -> 229      -0.21392

 Excited State   2:      Triplet-A      3.2398 eV  382.69 nm  f=n.d.  <S**2>=2.000
     225 -> 229      -0.17259
     225 -> 230      -0.13968
     225 -> 232       0.12774
     226 -> 229       0.16975
     226 -> 231      -0.10341
     226 -> 232       0.20432
     227 -> 231       0.20391
     227 -> 232      -0.11610
     228 -> 229      -0.20913
     228 -> 230       0.30360
     228 -> 232      -0.12019

 Excited State   3:      Triplet-A      3.2426 eV  382.36 nm  f=n.d.  <S**2>=2.000
     225 -> 229       0.26246
     225 -> 231       0.10699
     226 -> 230      -0.19245
     226 -> 232       0.13865
     227 -> 229      -0.15373
     227 -> 230       0.16094
     227 -> 231      -0.17053
     228 -> 230       0.10877
     228 -> 231       0.25709
     228 -> 232      -0.25456

 Excited State   4:      Triplet-A      3.2525 eV  381.20 nm  f=n.d.  <S**2>=2.000
     226 -> 230       0.15152
     226 -> 231       0.22224
     226 -> 232       0.21608
     227 -> 230       0.14042
     227 -> 231       0.24655
     227 -> 232       0.27687
     228 -> 231       0.17243
     228 -> 232       0.23006

 Excited State   5:      Triplet-A      3.9335 eV  315.20 nm  f=n.d.  <S**2>=2.000
     219 -> 232       0.10158
     220 -> 230      -0.11163
     222 -> 238       0.18235
     222 -> 241      -0.26612
     223 -> 235       0.16957
     223 -> 238       0.16783
     224 -> 233      -0.20355
     224 -> 235       0.22559

 Excited State   6:      Triplet-A      3.9389 eV  314.77 nm  f=n.d.  <S**2>=2.000
```

```
     217 -> 231       -0.10843
     221 -> 235       -0.10875
     221 -> 238        0.15267
     221 -> 241        0.10105
     222 -> 235       -0.17661
     222 -> 238       -0.13832
     223 -> 233        0.25603
     223 -> 241        0.19455
     224 -> 233       -0.13940
     224 -> 238       -0.14509

 Excited State   7:      Triplet-A      3.9432 eV  314.43 nm  f=n.d.  <S**2>=2.000
     218 -> 229        0.11505
     220 -> 232        0.10092
     221 -> 233        0.12596
     221 -> 235        0.17301
     221 -> 238       -0.15019
     222 -> 233        0.19601
     222 -> 235       -0.17525
     224 -> 235        0.11975
     224 -> 241        0.23208

 Excited State   8:      Triplet-A      3.9461 eV  314.19 nm  f=n.d.  <S**2>=2.000
     217 -> 229       -0.14849
     218 -> 230        0.11853
     221 -> 233        0.25649
     221 -> 235        0.22131
     221 -> 238       -0.11595
     222 -> 233       -0.11425
     223 -> 233        0.11535
     223 -> 238        0.18714
     224 -> 241       -0.15970

 Excited State   9:      Triplet-A      4.0753 eV  304.23 nm  f=n.d.  <S**2>=2.000
     217 -> 233       -0.13380
     218 -> 235        0.11570
     219 -> 235        0.16441
     219 -> 238        0.13420
     220 -> 238       -0.11138
     220 -> 241        0.21152
     222 -> 230        0.14821
     222 -> 258       -0.10209
     223 -> 231        0.11936
     224 -> 232        0.13883
     224 -> 249       -0.10620
     225 -> 233       -0.10818
     226 -> 235        0.12724

 Excited State  10:      Triplet-A      4.0827 eV  303.68 nm  f=n.d.  <S**2>=2.000
     217 -> 233       -0.17780
     217 -> 235       -0.11741
     218 -> 238       -0.17362
     219 -> 241        0.14187
     220 -> 238        0.11502
     221 -> 229        0.14773
     222 -> 231       -0.11685
     223 -> 250        0.10444
     225 -> 233       -0.12551

 Excited State  11:      Triplet-A      4.0915 eV  303.03 nm  f=n.d.  <S**2>=2.000
     217 -> 235       -0.11066
     217 -> 238        0.12997
     218 -> 233        0.17349
     218 -> 235        0.11561
     218 -> 241       -0.10973
     219 -> 241       -0.12404
     220 -> 235       -0.16272
     221 -> 229        0.10823
     221 -> 230        0.12194
     221 -> 250       -0.11217
     222 -> 232        0.10293
```

```
     224 -> 230         0.11807
     226 -> 241        -0.10718

 Excited State  12:      Triplet-A      4.0997 eV  302.42 nm  f=n.d.  <S**2>=2.000
     217 -> 238        -0.11613
     217 -> 241        -0.15412
     218 -> 233        -0.10157
     218 -> 235        -0.13022
     219 -> 233         0.14019
     219 -> 238         0.10534
     220 -> 233         0.15802
     220 -> 235        -0.10768
     222 -> 232         0.10781
     223 -> 229         0.10789
     223 -> 231         0.10812
     224 -> 229        -0.12078
     224 -> 230         0.10646
     225 -> 241        -0.12617

 Excited State  13:      Triplet-A      4.7728 eV  259.77 nm  f=n.d.  <S**2>=2.000
     222 -> 229        -0.10050
     223 -> 229         0.12216
     223 -> 231         0.12619
     225 -> 233         0.12336
     227 -> 233        -0.10296
     228 -> 241         0.11361

 Excited State  14:      Triplet-A      4.7756 eV  259.62 nm  f=n.d.  <S**2>=2.000
     222 -> 232         0.10250
     224 -> 230         0.11666
     225 -> 229         0.10855
     228 -> 233         0.13324

 Excited State  15:      Triplet-A      4.7940 eV  258.62 nm  f=n.d.  <S**2>=2.000
     213 -> 265        -0.10074
     221 -> 229         0.16299
     221 -> 230         0.15269
     225 -> 233         0.10194
     225 -> 235         0.11527
     225 -> 250         0.11641

 Excited State  16:      Triplet-A      4.7994 eV  258.33 nm  f=n.d.  <S**2>=2.000
     222 -> 231        -0.11821
     222 -> 232        -0.11993
     223 -> 231        -0.10409
     223 -> 232        -0.10502
     227 -> 253         0.10325

 Excited State  17:      Singlet-A      5.0307 eV  246.45 nm  f=0.0007  <S**2>=0.000
     225 -> 229        -0.28588
     226 -> 230         0.25260
     227 -> 229         0.11276
     227 -> 230        -0.14633
     227 -> 231         0.24489
     228 -> 231        -0.20897
     228 -> 232         0.31839

 Excited State  18:      Triplet-A      5.0350 eV  246.25 nm  f=n.d.  <S**2>=2.000
     220 -> 230        -0.10348
     222 -> 229         0.10278
     222 -> 230        -0.11983
     223 -> 229        -0.10178
     223 -> 231        -0.10286
     224 -> 230         0.14852
     224 -> 232        -0.17545

 Excited State  19:      Triplet-A      5.0372 eV  246.14 nm  f=n.d.  <S**2>=2.000
     223 -> 229        -0.15713
     223 -> 231        -0.15413
     224 -> 229         0.15383
     224 -> 230        -0.13463
```

```
Excited State  20:      Triplet-A      5.0550 eV  245.27 nm  f=n.d.  <S**2>=2.000
    218 -> 230        0.10489
    218 -> 233       -0.11080
    218 -> 235       -0.11394
    221 -> 229        0.25038
    221 -> 230        0.22114
    221 -> 231       -0.11746
    221 -> 271        0.10650

Excited State  21:      Triplet-A      5.0600 eV  245.03 nm  f=n.d.  <S**2>=2.000
    220 -> 235        0.10273
    222 -> 231        0.13904
    222 -> 232        0.16212
    223 -> 231        0.13807
    223 -> 232        0.14047
    224 -> 230        0.10614
    224 -> 231        0.13999
    224 -> 232        0.12272

Excited State  22:      Singlet-A      5.0611 eV  244.98 nm  f=0.0018  <S**2>=0.000
    225 -> 229       -0.19901
    225 -> 230       -0.20700
    226 -> 229        0.23545
    226 -> 231       -0.19481
    227 -> 230       -0.15639
    227 -> 232       -0.21185
    228 -> 229       -0.13031
    228 -> 230        0.24655
    228 -> 231       -0.13880
    228 -> 232       -0.18004

Excited State  23:      Triplet-A      5.0874 eV  243.71 nm  f=n.d.  <S**2>=2.000
    220 -> 232        0.11670
    224 -> 241       -0.10151
    226 -> 241       -0.12154
    228 -> 233       -0.13141
    228 -> 235        0.12682

Excited State  24:      Triplet-A      5.0903 eV  243.57 nm  f=n.d.  <S**2>=2.000
    219 -> 232       -0.11056
    220 -> 230        0.11474
    220 -> 231        0.11393
    226 -> 235        0.12665
    227 -> 238        0.10117
    227 -> 241       -0.10880

Excited State  25:      Triplet-A      5.0928 eV  243.45 nm  f=n.d.  <S**2>=2.000
    217 -> 229        0.10642
    217 -> 250        0.10182
    227 -> 233        0.11951

Excited State  26:      Singlet-A      5.0950 eV  243.34 nm  f=0.2046  <S**2>=0.000
    225 -> 230        0.19372
    225 -> 231       -0.24821
    225 -> 232        0.10840
    226 -> 229       -0.12589
    226 -> 230       -0.22046
    227 -> 229        0.31458
    227 -> 232       -0.13793
    228 -> 229       -0.25325
    228 -> 231       -0.13678

Excited State  27:      Triplet-A      5.1167 eV  242.31 nm  f=n.d.  <S**2>=2.000
    217 -> 229       -0.11118
    217 -> 230       -0.10587
    218 -> 229        0.15224
    218 -> 230        0.12921
    221 -> 250        0.11382
    221 -> 255        0.11009
    221 -> 256       -0.10258
```

```
     225 -> 233       -0.11186
     225 -> 235       -0.11555

 Excited State  28:      Singlet-A      5.1191 eV  242.20 nm  f=0.4661  <S**2>=0.000
     222 -> 241       -0.10228
     225 -> 232        0.10162
     226 -> 231        0.17643
     226 -> 232        0.28624
     227 -> 230        0.18590
     227 -> 231        0.26949
     227 -> 232        0.17994
     228 -> 229       -0.17352
     228 -> 230        0.24196
     228 -> 231        0.16951

 Excited State  29:      Triplet-A      5.2285 eV  237.13 nm  f=n.d.  <S**2>=2.000
     214 -> 262       -0.12080
     214 -> 263       -0.14470
     214 -> 265       -0.10938
     216 -> 262       -0.16034

 Excited State  30:      Triplet-A      5.2327 eV  236.94 nm  f=n.d.  <S**2>=2.000
     213 -> 265        0.12755
     214 -> 262        0.12779
     215 -> 263       -0.10128
     216 -> 262       -0.12998
     216 -> 263        0.11078
     216 -> 266        0.12562

 Excited State  31:      Triplet-A      5.2371 eV  236.74 nm  f=n.d.  <S**2>=2.000
     213 -> 261       -0.13052
     213 -> 263       -0.12452
     213 -> 265        0.24009
     214 -> 262       -0.11610

 Excited State  32:      Triplet-A      5.2410 eV  236.57 nm  f=n.d.  <S**2>=2.000
     196 -> 264        0.14659
     215 -> 262        0.11703
     215 -> 264       -0.26485
     215 -> 266       -0.12996
     216 -> 263        0.12296
     216 -> 264       -0.15540

 Excited State  33:      Triplet-A      5.2790 eV  234.86 nm  f=n.d.  <S**2>=2.000
     213 -> 229        0.18062
     213 -> 230        0.17790
     213 -> 231       -0.10574
     225 -> 261        0.11214
     225 -> 265       -0.18086
     226 -> 265        0.13023
     228 -> 263       -0.10831

 Excited State  34:      Triplet-A      5.2800 eV  234.82 nm  f=n.d.  <S**2>=2.000
     215 -> 232        0.10273
     216 -> 230        0.14961
     216 -> 232       -0.12559
     226 -> 262        0.12159
     226 -> 263       -0.11556
     228 -> 262       -0.12457
     228 -> 263        0.15030
     228 -> 265        0.10460
     228 -> 266        0.11997

 Excited State  35:      Triplet-A      5.2821 eV  234.73 nm  f=n.d.  <S**2>=2.000
     214 -> 229        0.15657
     214 -> 231        0.16851
     225 -> 263       -0.12811
     227 -> 265        0.11213
     228 -> 262       -0.16293

 Excited State  36:      Triplet-A      5.2892 eV  234.41 nm  f=n.d.  <S**2>=2.000
```

```
     207 -> 264        -0.10074
     215 -> 231         0.14843
     215 -> 232         0.15710
     216 -> 232         0.11812
     226 -> 264        -0.18178
     227 -> 264        -0.20911
     228 -> 264        -0.15483

 Excited State  37:      Singlet-A      5.4201 eV  228.75 nm  f=0.0002  <S**2>=0.000
     219 -> 231        -0.11140
     220 -> 232         0.14601
     222 -> 232         0.14939
     222 -> 233         0.12120
     223 -> 229         0.14878
     223 -> 231         0.16103
     223 -> 238        -0.11274
     224 -> 229        -0.17843
     224 -> 230         0.20050
     224 -> 241         0.17696
     225 -> 229         0.12960
     228 -> 233         0.15506

 Excited State  38:      Singlet-A      5.4489 eV  227.54 nm  f=0.0008  <S**2>=0.000
     221 -> 229        -0.11258
     221 -> 230        -0.10248
     222 -> 229        -0.10449
     222 -> 232        -0.10612
     223 -> 229         0.18985
     223 -> 232        -0.12783
     223 -> 241        -0.10605
     224 -> 230        -0.14724
     224 -> 231        -0.16095
     224 -> 238         0.11331
     227 -> 233        -0.13663
     228 -> 235         0.11873

 Excited State  39:      Singlet-A      5.4825 eV  226.15 nm  f=0.0098  <S**2>=0.000
     218 -> 229         0.12448
     221 -> 229         0.20008
     221 -> 230         0.15420
     221 -> 235         0.10972
     222 -> 231        -0.14144
     223 -> 230        -0.12845
     223 -> 232        -0.12426
     224 -> 231        -0.11174
     224 -> 232        -0.14109
     224 -> 235         0.10643
     226 -> 233        -0.10383
     227 -> 235         0.11768
     228 -> 238         0.10246

 Excited State  40:      Singlet-A      5.4945 eV  225.65 nm  f=0.4595  <S**2>=0.000
     220 -> 230         0.10192
     221 -> 229         0.14847
     221 -> 230         0.12673
     222 -> 230         0.15608
     223 -> 231         0.19820
     224 -> 232         0.20579
     224 -> 233         0.13547
     226 -> 235        -0.13887
     227 -> 238        -0.13850
     228 -> 241         0.13888

 Excited State  41:      Triplet-A      5.6655 eV  218.84 nm  f=n.d.  <S**2>=2.000
     223 -> 284        -0.10677
     225 -> 241        -0.10051
     228 -> 233        -0.11066

 Excited State  42:      Triplet-A      5.6673 eV  218.77 nm  f=n.d.  <S**2>=2.000
     226 -> 241        -0.10569
     228 -> 241         0.10902
```

Table S7 First TD-ωB97X/6-311++G(d,p) triplet and singlet electronic transitions computed for the tetramer comprising two π-π stacked H-bonded dimers of **TT-CCH** (Figure 6e) at the optimized ground state geometry.

```
Excitation energies and oscillator strengths:

 Excited State   1:      Triplet-A      3.2106 eV  386.17 nm  f=n.d.  <S**2>=2.000
     225 -> 230        0.36311
     225 -> 231        0.13979
     226 -> 229        0.41725
     226 -> 232        0.11811
     227 -> 230       -0.20932

 Excited State   2:      Triplet-A      3.2109 eV  386.13 nm  f=n.d.  <S**2>=2.000
     225 -> 229        0.38190
     226 -> 230        0.42059
     226 -> 231        0.12541
     227 -> 229       -0.18177

 Excited State   3:      Triplet-A      3.2212 eV  384.90 nm  f=n.d.  <S**2>=2.000
     225 -> 232        0.16921
     227 -> 229       -0.13077
     227 -> 232        0.36476
     228 -> 230       -0.13207
     228 -> 231        0.41485

 Excited State   4:      Triplet-A      3.2234 eV  384.63 nm  f=n.d.  <S**2>=2.000
     225 -> 230       -0.11685
     225 -> 231        0.14557
     227 -> 231        0.39339
     228 -> 229       -0.11875
     228 -> 232        0.39535

 Excited State   5:      Triplet-A      3.9412 eV  314.58 nm  f=n.d.  <S**2>=2.000
     219 -> 232       -0.14977
     220 -> 231        0.13139
     223 -> 234       -0.19911
     223 -> 239        0.24255
     223 -> 240        0.21201
     224 -> 233        0.22686
     224 -> 238        0.28465

 Excited State   6:      Triplet-A      3.9432 eV  314.43 nm  f=n.d.  <S**2>=2.000
     219 -> 231       -0.14012
     220 -> 232        0.13336
     223 -> 233        0.22850
     223 -> 238        0.29984
     224 -> 234       -0.18581
     224 -> 239        0.23161
     224 -> 240        0.20447

 Excited State   7:      Triplet-A      3.9611 eV  313.01 nm  f=n.d.  <S**2>=2.000
     217 -> 229       -0.11306
     218 -> 229       -0.10345
     218 -> 230       -0.11910
     221 -> 233       -0.18341
     221 -> 234        0.25793
     221 -> 238        0.13368
     222 -> 233        0.20658
     222 -> 234       -0.21344
     222 -> 238       -0.14964

 Excited State   8:      Triplet-A      3.9611 eV  313.01 nm  f=n.d.  <S**2>=2.000
     217 -> 230        0.11460
     218 -> 229        0.11828
     218 -> 230       -0.10484
     221 -> 233        0.23577
     221 -> 234        0.18152
     221 -> 238       -0.14625
     222 -> 233        0.21770
```

```
     222 -> 234         0.22924
     222 -> 238        -0.13460
     222 -> 240         0.10062

 Excited State   9:      Triplet-A      4.0987 eV  302.49 nm  f=n.d.  <S**2>=2.000
     217 -> 233        -0.13089
     219 -> 234         0.16068
     219 -> 239        -0.17676
     219 -> 240        -0.14956
     220 -> 233         0.10526
     220 -> 238         0.21890
     223 -> 232         0.18625
     224 -> 231         0.17504
     224 -> 255        -0.10792

 Excited State  10:      Triplet-A      4.0989 eV  302.48 nm  f=n.d.  <S**2>=2.000
     217 -> 234         0.13106
     219 -> 233        -0.15379
     219 -> 238        -0.21790
     220 -> 234        -0.10122
     220 -> 239         0.17466
     220 -> 240         0.15161
     223 -> 231         0.18182
     223 -> 255        -0.12634
     224 -> 232         0.16221

 Excited State  11:      Triplet-A      4.1131 eV  301.44 nm  f=n.d.  <S**2>=2.000
     217 -> 233        -0.18470
     217 -> 238         0.16768
     218 -> 234         0.23409
     218 -> 239         0.10036
     218 -> 240         0.10093
     220 -> 233        -0.16375
     221 -> 230        -0.17679
     221 -> 252        -0.13766
     222 -> 229         0.18537
     222 -> 248         0.11462
     226 -> 234         0.10184

 Excited State  12:      Triplet-A      4.1131 eV  301.44 nm  f=n.d.  <S**2>=2.000
     217 -> 234        -0.19224
     217 -> 239        -0.11350
     217 -> 240        -0.11032
     218 -> 233         0.23005
     218 -> 238        -0.15150
     220 -> 234        -0.15538
     221 -> 229         0.18356
     221 -> 248         0.11731
     222 -> 230        -0.17821
     222 -> 252        -0.13609

 Excited State  13:      Triplet-A      4.7658 eV  260.15 nm  f=n.d.  <S**2>=2.000
     211 -> 232         0.13299
     212 -> 231         0.10109
     223 -> 232         0.11373
     224 -> 231         0.16428
     227 -> 232        -0.12044
     227 -> 238         0.14549
     227 -> 248        -0.13507
     228 -> 234        -0.11558
     228 -> 239         0.10447
     228 -> 255         0.13772
     228 -> 275         0.11029

 Excited State  14:      Triplet-A      4.7673 eV  260.07 nm  f=n.d.  <S**2>=2.000
     211 -> 231         0.11452
     212 -> 232         0.11221
     224 -> 232         0.16408
     227 -> 230         0.11332
     227 -> 234        -0.10478
     227 -> 239         0.11715
```

```
     227 -> 255         0.12916
     227 -> 275         0.10239
     228 -> 238         0.13656
     228 -> 248        -0.13169

 Excited State  15:      Triplet-A      4.7871 eV  259.00 nm  f=n.d.  <S**2>=2.000
     208 -> 230         0.12273
     221 -> 230         0.15226
     222 -> 229        -0.14391
     225 -> 233        -0.11632
     225 -> 238         0.11066
     225 -> 248         0.14015
     226 -> 234         0.17143
     226 -> 252         0.17585
     226 -> 270         0.10334

 Excited State  16:      Triplet-A      4.7884 eV  258.93 nm  f=n.d.  <S**2>=2.000
     208 -> 229        -0.11579
     221 -> 229        -0.14661
     222 -> 230         0.13961
     225 -> 230        -0.10191
     225 -> 234        -0.13267
     225 -> 252        -0.14496
     226 -> 233         0.11991
     226 -> 238        -0.13167
     226 -> 248        -0.14303
     227 -> 234         0.11804

 Excited State  17:      Singlet-A      4.9960 eV  248.16 nm  f=0.0000  <S**2>=0.000
     225 -> 229        -0.28782
     226 -> 230        -0.27601
     227 -> 232         0.33822
     228 -> 230        -0.12844
     228 -> 231         0.35201

 Excited State  18:      Triplet-A      5.0347 eV  246.26 nm  f=n.d.  <S**2>=2.000
     217 -> 234         0.14331
     218 -> 229         0.12022
     218 -> 233        -0.12798
     221 -> 229         0.26746
     221 -> 272         0.10901
     222 -> 230        -0.25401
     222 -> 270        -0.12717

 Excited State  19:      Triplet-A      5.0349 eV  246.25 nm  f=n.d.  <S**2>=2.000
     217 -> 233         0.12435
     218 -> 230        -0.11999
     218 -> 234        -0.15757
     218 -> 270        -0.10846
     221 -> 230        -0.25600
     221 -> 270        -0.12933
     222 -> 229         0.26526
     222 -> 272         0.10652
     226 -> 252         0.10696

 Excited State  20:      Singlet-A      5.0368 eV  246.16 nm  f=0.3358  <S**2>=0.000
     225 -> 230         0.39417
     226 -> 229         0.43374
     226 -> 232         0.12735
     227 -> 230        -0.18097
     227 -> 231        -0.11657

 Excited State  21:      Triplet-A      5.0471 eV  245.65 nm  f=n.d.  <S**2>=2.000
     219 -> 238         0.14012
     220 -> 234         0.10011
     223 -> 230        -0.10127
     223 -> 231         0.28064
     224 -> 229        -0.10231
     224 -> 232         0.24939

 Excited State  22:      Triplet-A      5.0493 eV  245.55 nm  f=n.d.  <S**2>=2.000
```

```
    219 -> 234       -0.10302
    220 -> 232        0.12159
    220 -> 238       -0.10802
    223 -> 229       -0.10735
    223 -> 232        0.23825
    224 -> 231        0.27778

Excited State  23:      Triplet-A      5.0633 eV  244.87 nm  f=n.d.  <S**2>=2.000
    219 -> 229        0.10498
    219 -> 232       -0.15167
    220 -> 231        0.17268
    223 -> 255        0.11512
    228 -> 233       -0.14454
    228 -> 238       -0.19209

Excited State  24:      Triplet-A      5.0651 eV  244.78 nm  f=n.d.  <S**2>=2.000
    219 -> 231       -0.16410
    219 -> 255        0.11298
    220 -> 232        0.12430
    220 -> 233        0.10654
    220 -> 238        0.10014
    223 -> 238       -0.11490
    227 -> 238       -0.11187
    228 -> 234        0.10786
    228 -> 239       -0.15406
    228 -> 240       -0.13716

Excited State  25:      Singlet-A      5.0729 eV  244.41 nm  f=0.0000  <S**2>=0.000
    225 -> 229        0.26413
    225 -> 232        0.18642
    226 -> 230        0.33384
    227 -> 229       -0.24095
    227 -> 232        0.17088
    228 -> 230       -0.11691
    228 -> 231        0.24471

Excited State  26:      Singlet-A      5.0905 eV  243.56 nm  f=0.8068  <S**2>=0.000
    225 -> 231        0.18304
    227 -> 230       -0.18112
    227 -> 231        0.37785
    228 -> 229       -0.14406
    228 -> 232        0.40807

Excited State  27:      Triplet-A      5.1120 eV  242.53 nm  f=n.d.  <S**2>=2.000
    217 -> 229        0.16755
    217 -> 233        0.10120
    218 -> 230        0.18285
    221 -> 252       -0.11860
    222 -> 233        0.10501
    225 -> 233        0.16701
    226 -> 234       -0.18535

Excited State  28:      Triplet-A      5.1125 eV  242.51 nm  f=n.d.  <S**2>=2.000
    217 -> 230        0.16868
    218 -> 229        0.18624
    218 -> 233        0.10420
    221 -> 233       -0.10421
    222 -> 252        0.12172
    225 -> 234       -0.16501
    226 -> 233        0.18656
    226 -> 238       -0.11429

Excited State  29:      Triplet-A      5.2117 eV  237.90 nm  f=n.d.  <S**2>=2.000
    215 -> 261       -0.11632
    215 -> 265       -0.23091
    216 -> 259       -0.14300
    216 -> 260        0.11199
    216 -> 264        0.18573

Excited State  30:      Triplet-A      5.2133 eV  237.82 nm  f=n.d.  <S**2>=2.000
    215 -> 259       -0.14214
```

```
      215 -> 260         0.11273
      215 -> 264         0.18735
      216 -> 261        -0.11441
      216 -> 265        -0.23065

 Excited State  31:      Triplet-A      5.2532 eV  236.02 nm  f=n.d.  <S**2>=2.000
      213 -> 231        -0.12563
      214 -> 229         0.10843
      215 -> 230        -0.12731
      215 -> 231         0.13242
      216 -> 232         0.17130
      227 -> 261        -0.11019
      227 -> 265        -0.20347
      228 -> 259        -0.12611
      228 -> 264         0.13687
      228 -> 271        -0.10320

 Excited State  32:      Triplet-A      5.2567 eV  235.86 nm  f=n.d.  <S**2>=2.000
      213 -> 229         0.10388
      214 -> 231        -0.12025
      215 -> 232         0.17087
      216 -> 230        -0.12303
      216 -> 231         0.14223
      226 -> 265        -0.10390
      227 -> 259        -0.14005
      227 -> 260         0.10546
      227 -> 264         0.16402
      228 -> 265        -0.18204
      228 -> 273         0.10103

 Excited State  33:      Triplet-A      5.2874 eV  234.49 nm  f=n.d.  <S**2>=2.000
      213 -> 259         0.11242
      213 -> 260        -0.10109
      213 -> 264        -0.16427
      214 -> 265         0.19958
      215 -> 271        -0.10069
      215 -> 282         0.12041
      216 -> 273         0.12017

 Excited State  34:      Triplet-A      5.2884 eV  234.44 nm  f=n.d.  <S**2>=2.000
      213 -> 265         0.19439
      214 -> 259         0.11666
      214 -> 264        -0.16624
      215 -> 273         0.12067
      216 -> 282         0.12026

 Excited State  35:      Triplet-A      5.2948 eV  234.16 nm  f=n.d.  <S**2>=2.000
      213 -> 229         0.13818
      214 -> 230        -0.17369
      215 -> 229         0.17331
      216 -> 230        -0.11788
      216 -> 231        -0.13200
      225 -> 259         0.11260
      225 -> 260        -0.10374
      225 -> 264        -0.17103
      226 -> 265        -0.17602

 Excited State  36:      Triplet-A      5.2973 eV  234.05 nm  f=n.d.  <S**2>=2.000
      213 -> 230        -0.16826
      214 -> 229         0.14264
      215 -> 230        -0.12215
      215 -> 231        -0.13475
      216 -> 229         0.16566
      225 -> 265         0.19498
      226 -> 264         0.15739

 Excited State  37:      Singlet-A      5.4420 eV  227.83 nm  f=0.0000  <S**2>=0.000
      219 -> 231        -0.13321
      220 -> 232         0.14424
      223 -> 229        -0.11310
      223 -> 232         0.17953
```

```
    223 -> 233        0.19390
    223 -> 238        0.17508
    224 -> 231        0.27149
    224 -> 239        0.11936
    224 -> 240        0.11477
    225 -> 238        0.10060
    228 -> 231        0.12530
    228 -> 234       -0.11822
    228 -> 239        0.14137
    228 -> 240        0.12336

Excited State  38:      Singlet-A      5.4635 eV  226.93 nm  f=0.1241  <S**2>=0.000
    219 -> 232       -0.11951
    220 -> 231        0.15112
    221 -> 229        0.11835
    222 -> 230       -0.12440
    223 -> 231        0.24205
    223 -> 239        0.14449
    223 -> 240        0.13732
    224 -> 229       -0.12252
    224 -> 232        0.18786
    224 -> 233        0.14365
    224 -> 238        0.11937
    228 -> 233        0.16185
    228 -> 238        0.17940

Excited State  39:      Singlet-A      5.4830 eV  226.12 nm  f=0.4785  <S**2>=0.000
    217 -> 230        0.11624
    218 -> 229        0.15683
    220 -> 230        0.12842
    221 -> 229        0.23149
    221 -> 233        0.18125
    222 -> 230       -0.21338
    222 -> 231       -0.12825
    222 -> 234        0.14350
    225 -> 234        0.16600
    226 -> 233       -0.15939
    226 -> 238        0.10195
    228 -> 238       -0.10121

Excited State  40:      Singlet-A      5.4897 eV  225.85 nm  f=0.0000  <S**2>=0.000
    217 -> 229        0.12428
    218 -> 230        0.16332
    220 -> 229        0.13252
    221 -> 230        0.23644
    221 -> 231        0.11909
    221 -> 234       -0.13232
    222 -> 229       -0.23552
    222 -> 233       -0.18836
    224 -> 231       -0.10943
    225 -> 233       -0.15157
    226 -> 234        0.16783

Excited State  41:      Triplet-A      5.6259 eV  220.38 nm  f=n.d.  <S**2>=2.000
    215 -> 265       -0.10770
    225 -> 234       -0.10820
    227 -> 239        0.12309
    227 -> 240        0.10650
    228 -> 233        0.12496
    228 -> 238        0.14692

Excited State  42:      Triplet-A      5.6301 eV  220.22 nm  f=n.d.  <S**2>=2.000
    216 -> 265        0.10877
    225 -> 238       -0.11420
    227 -> 233       -0.14123
    227 -> 238       -0.10091
    228 -> 239       -0.11600
    228 -> 240       -0.10021
```
